# Evaluating PEACHES: Pleasure-Inclusive Implementation Strategies to Enable Healthcare Workers to Address Anal Sex Stigma During HIV Service Encounters

**DOI:** 10.1007/s10508-026-03500-7

**Published:** 2026-08-19

**Authors:** Bryan A. Kutner, Long-Jie Huang, Charlotte E. Rinnooy Kan, Baichun Hou, Yingchen Xu, Rebecca Giguere, Jim Pickett, Mitchell J. Wharton, Sara Bowen-Lasisi, Edgar Quintero, Wenzhu Bi Mowrey, Theodorus G. M. Sandfort, members of The Pleasure, Health Study Advisory Board

**Affiliations:** 1 Department of Psychiatry and Behavioral Sciences, Albert Einstein College of Medicine and Montefiore Medical Center, 1225 Morris Park Ave., Suite 4A, Bronx, NY 10461 USA; 2 Department of Educational and Counseling Psychology, University at Albany, State University of New York, Albany, NY USA; 3 Department of Biological Sciences, School of Arts and Sciences, Columbia University Graduate, New York, NY USA; 4 Department of Family and Community Medicine, University of San Franciso, San Francisco, CA USA; 5 Department of Epidemiology & Population Health, Albert Einstein College of Medicine, Bronx, NY USA; 6 Center for Translational Behavioral Science, Behavioral Science at Florida State University, Tallahassee, FL USA; 7 Jim Pickett Consulting, Chicago, IL USA; 8 Us Helping Us, People Into Living, Inc., Washington, DC USA; 9 Springfield Urban League, Springfield, IL USA; 10 HIV Center for Clinical and Behavioral Studies at Columbia University and New York State Psychiatric Institute, New York, NY USA

**Keywords:** Anal sex stigma, COM-B model, HIV prevention, Social stigma, Sexual orientation

## Abstract

Stigma toward anal sex hinders HIV prevention by limiting equitable access to sexual health services. To mitigate this hindrance, we developed a clinician- and community-informed package of anti-stigma implementation strategies using the Behaviour Change Wheel and its Capability, Opportunity, Motivation, Behaviour (COM-B) Model. The package, “Partnering to Enhance Anal Health Communication and HIV-related Evidence-based Services” (PEACHES 1.0), combined training strategies with quality improvement (QI) strategies. We implemented a pilot evaluation of PEACHES 1.0 among HIV workers in the Southern USA and assessed pre/post-shifts in participants’ self-reported proximal outcomes (mechanisms of action like knowledge, comfort, skills) and their frequency of anal sex-specific health promotion. In two clinics that received organization-specific QI meetings, we also assessed patient uptake of anal sex-related HIV services based on aggregated medical record data. We conducted paired *t*-tests on longitudinal survey responses, thematic analysis of longitudinal in-depth interviews and open-ended survey responses, and non-statistical comparison of rates for patient uptake of services. Post-PEACHES, HIV workers reported 8% to 54% improvements (all *p* < .01) in proximal outcomes like knowledge, comfort, and skills. Frequency of self-reported anal health promotion increased 15% to 25% (*p* < .01). Clinics that received QI sessions differed in their QI engagement and had diverging patient uptake of services. Clinic A, with greater QI engagement, saw a 60% increase in anorectal STI screening, and a 28% increase in new PrEP prescriptions; Clinic B, with less engagement, saw 17% and 18% decreases, respectively. Qualitative findings indicated that the QI strategies—placing pleasure-inclusive visual cues linked to a website of informational resources in the clinic environment and clinic-specific QI meetings—enabled workers to destigmatize discussions about anal sex within HIV services. Future research should standardize elements of QI, and evaluate scalability with fidelity as well as the potential impact on client outcomes in a fully-powered hybrid implementation-effectiveness trial.

**Supplementary Information:**

The online version contains supplementary material available at https://doi.org/10.1007/s10508-026-03500-7.

## Introduction

Anal intercourse is a prevalent and highly stigmatized sexual behavior (Ayres & Luedman, 2013; Benson et al., 2019; Branfman et al., 2017; Exner et al., 2008; Faustino, 2020, 2021; Fields et al., 2012; Halperin, 1999; Hoppe, 2011; Katz et al., 2023; Kutner et al., 2020a, 2020b, 2020c, 2020d; McBride, 2019; McDavitt & Mutchler, 2014; Quinn et al., 2019; Ravenhill & Visser, 2018; Roye et al., 2010, 2013; Winder, 2023). Stigma toward anal sex is rooted in homophobia and social norms that frame heterosexual penile-vaginal intercourse as natural, and deviations from reproductive sex as immoral (Tyler & Slater, 2018; Weiss, 2008). As a result of stigma, healthcare workers (HCWs) often neglect to discuss anal sex with patients, which is particularly problematic in HIV prevention and care settings, where assessment of sexual behavior often precedes access to interventions that can protect against HIV and other sexually transmitted infections (STIs) (Ayala et al., 2013; Gana & Hunt, 2022; Golub, 2018; Hebert et al., 2017; Kutner et al., 2019, 2022, 2024; Nadarzynski et al., 2018; Sam et al., 2025; Turpin et al., 2025; Wall et al., 2010). For example, anal sex can be stereotyped as occurring primarily between men, despite evidence that more women than men have engaged in receptive penile-anal intercourse (Copen et al., 2016; Herbenick et al., 2017). This stereotype of anal sex as “gay sex” can lead HCWs to neglect sexual health screenings in women. Indeed, in a medical records study of 7.8 million women who had tested for STIs in the USA, < 0.1 percent received anorectal screenings (Tao et al., 2018). This missed opportunity is particularly troubling because anorectal infections render mucosa more susceptible to HIV (Fleming & Wasserheit, 1999; Tao et al., 2018), and unprotected anal intercourse is responsible for an estimated 28–40% of new cases of HIV among women (Elmes et al., 2020; Stannah et al., 2020)—as well as nearly all cases among men and many transgender people (Sullivan et al., 2009).

Gaps in the provision of anal sex-related health services by HCWs can also be credited to providers’ lack of knowledge, skill, and comfort communicating holistically about anal sex (Dickstein et al., 2024; Gana & Hunt, 2022; Kutner et al., 2020a). The World Health Organization defines sexual health broadly to include pleasure rather than simply freedom from pathology (WHO, 2006), and a recent systematic review suggests that incorporating pleasure within sexual health interventions adds moderate value (Cohen’s *d* = 0.37) to increasing condom usage (Zaneva 2022). Yet, sexual health providers are typically trained to focus on disease identification and treatment, which can unwittingly reinforce stereotypes and stigma around anal sex (Morin, 2010). When sexual health discussions focus primarily on infectious disease, patients may conceal stigmatized sexual behavior out of shame or anticipation of mistreatment (Brooks et al., 2018; Kutner et al., 2020a; Qiao et al., 2018; Rispel et al., 2011; Verrastro et al., 2020). Holistic patient concerns about anal sex like pain, continence, contact with fecal matter, and pleasure are rarely discussed in healthcare settings (Chen & Kalichman, 2024; Collier et al., 2015; Dickstein et al., 2024; Exner et al., 2008; Kutner et al., 2019; Kutner et al., 2020a; Sandfort & Keizer, 2001). Avoidance of these topics by providers and concealment by patients interfere with providers’ ability to conduct sexual histories and deliver anal sex-related evidence-based interventions—like screening and treatment for anorectal STIs and HIV pre-exposure prophylaxis (PrEP)—that could otherwise reduce, if not entirely eliminate, the risk of HIV transmission during anal sex (Bernstein et al., 2008; Brooks et al., 2018; Fisher et al., 2018; Kutner et al., 2020a, 2020b, 2020c, 2020d; Mayer et al., 2012; Meites et al., 2013; Qiao et al., 2018; Singh et al., 2018).

Training HCWs to communicate knowledgeably, skillfully, and comfortably about anal sex may increase engagement in HIV and sexual health services. From the patient perspective, comfort talking about anal sex with providers and sources of informational and emotional social support around anal sex have been associated with greater uptake of anal sex-related HIV services (Kutner et al., 2020a, 2020b, 2020c, 2020d). Yet few professional training programs equip providers to communicate about anal sex holistically in ways that increase patient comfort and access to social support (Prize et al., 2023). In 2017, we conducted a pilot evaluation of a workshop program in China to increase health worker comfort discussing anal sexuality in HIV services, entitled “Smarter Sex is the New Safer Sex: Understanding Anal Pleasure and Health for the HIV Workforce.” HCWs in China found the workshop to be highly acceptable, feasible, and appropriate (Kutner et al., 2019) and reported that it increased their knowledge, skill, and comfort discussing anal sex (Kutner et al., 2025). Primary recommendations for improving the workshop included adding patient-facing visual and educational strategies (e.g., pamphlets and online materials) to combat anal sex stigma in clinical environments as well as online components to allow patients to access informational and emotional support online (Kutner et al., 2019).

**Fig. 1:**
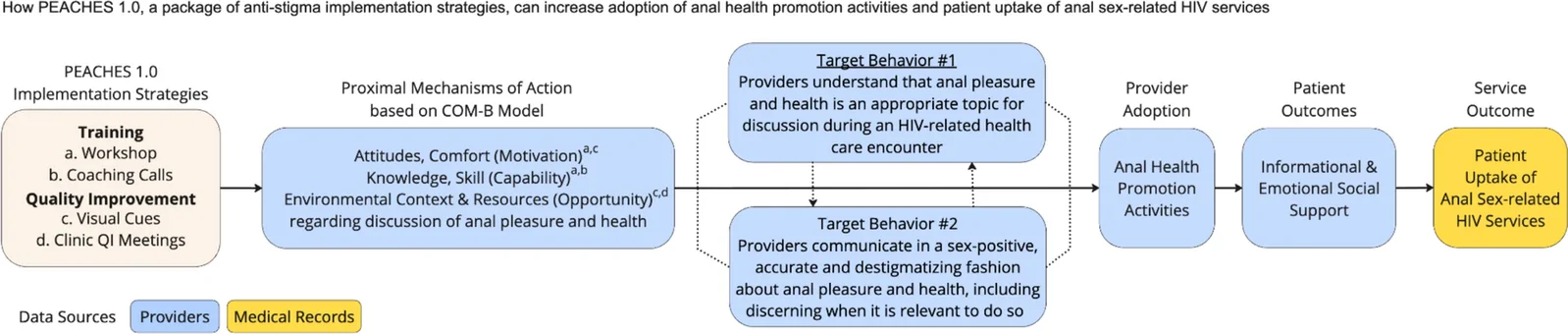
Conceptual model for implementation strategy components within the PEACHES 1.0 package

To advance our understanding of how to promote holistic discussion about anal health and assess the impact of this promotion on patient access to anal sex-related HIV services, we developed and pilot tested a package of anti-stigma strategies (PEACHES 1.0, Partnering to Enhance Anal health Communication and HIV-related Evidence-based Services). To develop the package, we applied the multistep *Behaviour Change Wheel* process and its *COM-B Model (Capability, Opportunity, Motivation and Behaviour)* (Michie et al. 2014; West and Michie 2020) by synthesizing recommendations from patients and providers based on interviews, surveys, and advisory board consultations. Our conceptual model, as shown in Fig. 1, depicts how PEACHES 1.0 aims to stimulate shifts in HCWs’ attitudes and comfort (motivation), knowledge and skills (capability), and environmental context and resources (opportunity) to discuss anal pleasure and health. Changes in these mechanisms of action (proximal outcomes in Fig. 1) are intended to influence two more distal target behaviors: HCWs’ understanding that the topic is appropriate during a healthcare encounter (Target Behavior 1) and HCWs’ ability to communicate about anal sex in an accurate and sex-positive way (Target Behavior 2). Provider adoption of these behaviors is posited to increase patient comfort and social support, leading to increased uptake of anal sex-related HIV services.

## Method

### Provider-Level Training and Clinic-level Quality Improvement Strategies: PEACHES 1.0

The multi-level anti-stigma package, PEACHES 1.0 (hereafter PEACHES), comprised two strategies: provider-level training and clinic-level quality improvement (QI). PEACHES was delivered twice, initially in Tennessee (Region 1) followed by Alabama and Mississippi (Region 2), each time over the course of 3.5 months. PEACHES comprised four activities, two training activities and two QI activities.

Activity 1: Training began with an updated version of the two-day, in-person “Smarter Sex” workshop for health workers that had been delivered in China, to clarify values and increase knowledge, skills, and comfort discussing anal sex. The workshop combined interactive content on anal anatomy, physiology, and sexual response with communication skills based on Motivational Interviewing (e.g., the information exchange skill of Ask-Offer-Ask) (Miller & Rollnick, 2023).

Activity 2: Training continued with four cross-site coaching calls to reinforce knowledge and skills from the workshop.

Activity 3: QI activities began when workshop participants received patient-facing visual cues for placement in their clinical environments, such as certificates of workshop completion, posters, palm cards, peach and prostate-shaped stress balls. All materials directed patients to pleasureandhealth.org, a project-developed website of accurate, reliable, sex-positive patient-facing information about anal pleasure and health.

All participants received Activity 1 and were offered opportunities to participate in Activities 2 and 3.

Activity 4: Participants from the workshop at two clinics (Clinic A in Tennessee and Clinic B in Mississippi) also received organization-specific QI sessions for the development of policies and procedures, such as standard operating procedures for anorectal STI screening and revisions to intake forms. Leadership committed to set aside dedicated time for QI and sharing longitudinal, de-identified, aggregated medical record data on patient service outcomes, as posited in Fig. 1. To facilitate region-specific adaptation and ensure alignment with organizational priorities, the principal investigator (BAK) conducted pre-implementation interviews with site leaders of these selected organizations.

Supplemental File 1 specifies components of PEACHES according to Proctor’s Framework (Proctor et al., 2013) in terms of actors, actions, targets, temporality, dose, outcomes, and theoretical justification. Supplemental File 2 includes a categorization of each activity in terms of behavioral ontologies based on the BCW. This includes their general intervention types (or functions) as well as related behavior change techniques (BCTs), their smallest unit of intervention, based on the Behaviour Change Techniques Taxonomy version 1 (Human Behaviour Change Project: University College London 2014), and Behaviour Change Intervention Ontology (BCIO) (Human Behaviour Change Project, 2025). This categorization aims to facilitate eventual comparison and synthesis of our findings with other studies (Hennessy et al., 2019).

### Participants

Participants were HCWs responsible for the delivery, referral, or management of HIV services in either Tennessee (Region 1) or Mississippi/Alabama (Region 2). To match “real-world” implementation, the study team partnered with regional offices of the Southeast AIDS Education and Training Center (Southeast AETC) to host the workshop and to recruit one organization in each region for site-specific QI (Activity 4). The Southeast AETC distributed an IRB-approved flyer describing the workshop and research study. Interested health workers registered online and the Southeast AETC provided a roster of registrant email addresses to the study team. Prior to the workshop, registrants completed a REDCap survey of eligibility criteria, an information statement, and a baseline survey hosted by Albert Einstein College of Medicine (Harris et al., 2009, 2019). Entry into the *Smarter Sex* workshop required consent to participate in the study; data collection was voluntary. We recruited participants across 18 organizations and 19 distinct healthcare settings.

### Measures and Procedure

To understand the impact of PEACHES, we assessed self-reported changes in mechanisms of action (MOA, Fig. 1) for Target Behavior 1 and, for Target Behavior 2, frequency of anal health promotion activities. We also piloted the collection of medical record reports of patient uptake of services, with the goal of informing the design of future evaluation of PEACHES’ impact using client-level data for an interrupted time series analysis (Zhang et al., 2020).

Longitudinal assessments were conducted between November 2023 and February 2024 in Tennessee and between May and September 2024 in Mississippi/Alabama. In each region, REDCap surveys (including open-ended text-entry response items) assessed constructs across three timepoints: “Pre-PEACHES” (Timepoint 1); “Post-Workshop” (Timepoint 2); and “Post-PEACHES” (Timepoint 3, after we completed delivery of all four activities). Interviews were conducted via Zoom Pro Post-Workshop and Post-PEACHES by [BH]. Periodic debriefs after interviews were supervised by [RG] rather than the public-facing purveyor of PEACHES [BAK] to minimize social desirability bias. At the end of the study, we requested monthly medical record data from each of the two sites that received organization-specific QI sessions (Activity 4). This medical record data comprised 9 months of aggregated patient-level data, including three 3-month periods for pre-implementation, implementation, and post-implementation of PEACHES.

Pre-implementation interview participants received $100. PEACHES participants received $30 per completed survey and $50 per interview, and each organization received $500 for reporting medical record data. In each region, we conducted interviews until these ceased to yield new information (Guest et al., 2006).

All participants completed Pre-PEACHES surveys. Nearly all also completed Post-Workshop surveys (98.5%) and again Post-PEACHES (95.4%). Following the workshop, we conducted interviews with 28 participants (43.1%). After the completion of PEACHES, 19 of these participants, along with 5 additional participants, completed interviews, representing 36.9% of the total sample. Nearly all participants (64/65) contributed to qualitative inquiry, whether through interviews or responses to open-ended survey questions.

#### Measurement

##### Quantitative Assessment

**A. Mechanisms of Action (Target Behavior 1)**

**Knowledge.** The 14-item Inventory of Anal Sex Knowledge (iASK) assessed participants’ knowledge of anal pleasure and health. Response options for “False” and “I Don’t Know” were coded as “incorrect” to yield a percent correct (Kutner et al., 2022). The scale demonstrated low internal reliability (Cronbach’s α = .62).

**Skills; Positive and Negative Emotions; Professional Role and Identity; Self-efficacy; Informational Resources; Organizational Context and Resources.** The Determinants of Implementation Behaviour Questionnaire (DIBQ) (Huijg et al., 2014) measures multiple MOAs from the Theoretical Domains Framework (Atkins et al., 2017), the precursor to the Behaviour Change Wheel and subsequent BCIO ontologies (Human Behaviour Change Project, 2025). The *DIBQ* was designed to be tailored to the study context, allowing for assessment of a subset of relevant domains or MOAs, and was previously adapted to evaluate the Smarter Sex workshop in China (Kutner et al., 2025). The 26-item measure comprised seven subscales and all but one demonstrated very strong internal reliability: (1) Skills (Cronbach’s *α* = .91); (2) Positive Emotions (Cronbach’s *α* = .96); (3) Negative Emotions (Cronbach’s *α* = .88); (4) Professional Role and Identity (Cronbach’s *α* = .86); (5) *Self-efficacy* (Cronbach’s *α* = .93); (6) Informational Resources (Cronbach’s *α* = .80); and (7) Organizational Context and Resources (Cronbach’s *α* = .39). Likert response options varied across subscales, but all ranged from 1 to 6, with higher numbers indicating greater positive valence with regard to that mechanism, with the exception of the inverse for *Negative Emotions*.

**Confidence Offering Informational and Emotional Social Support**. The 8-item informational and emotional social support subscale of the Medical Outcomes Study Social Support Scale (MOS-SSS) (Sherbourne & Stewart, 1991) assessed HCWs’ ease/confidence supporting clients (“How easy would it be for you to support clients in the following ways?”), was based on a previously successful adaptation (Kutner et al., 2020a, 2020b, 2020c, 2020d), and demonstrated high reliability (Cronbach’s *α* = .97). Likert response options ranged from 1 (Very Difficult) to 5 (Very Easy).

**Comfort with Anal Health Assessment and Promotion**. We augmented a previously piloted measure of comfort with anal health assessment and promotion (Kutner et al., 2025), adding items to arrive at a 4-item measure of assessment and an 8-item measure of promotion. Likert response options ranged from 0 (Not at all Comfortable) to 4 (Extremely Comfortable).

##### B. Impact on Behaviour (Target Behavior 2)

***Frequency of Anal Health Assessment and Promotion.*** For each item from the measure of “Comfort with Anal Health Assessment and Promotion”, we assessed frequency of assessment and promotion (Kutner et al., 2025). Likert response options ranged from 0 (Not at all) to 4 (Always).

**Service Delivery.** From the two clinics that received site-specific QI meetings (Activity 4), we requested aggregated monthly data of the number of patients who received (1) anorectal, oropharyngeal, and/or urethral STI screening; (2) new PrEP prescriptions; and (3) HIV testing. Clinic A (Region 1) abstracted data from its electronic health record system; Clinic B (Region 2) provided handwritten tallies.

**Quantitative Analysis. **Demographic characteristics were summarized at the baseline period. Means and standard deviations were calculated for continuous variables, and counts and percentages for categorical variables. Paired t-tests assessed the change in continuous measures between timepoints, and McNemar’s tests assessed the percentage change in categorical measures between timepoints. All analyses were performed using R (version 4.4.1) (R Core Team, 2023), and statistical significance was defined as *p* < 0.05. No statistical analyses were performed on clinics’ patient service outcomes because clinic-level data were aggregated by month, not by patient. Data were used for an exploratory, descriptive comparison, rather than for a formal interrupted time series analysis.

### Qualitative Analysis

Open-ended questions from surveys and structured interview guides explored the impact of strategies on additional MOAs based on our conceptual model (i.e., beliefs about consequences, intentions toward addressing anal pleasure and health with clients), as well as HCW behavior, service quality, and client engagement.

Audio-recorded in-depth interviews were transcribed verbatim, reviewed for accuracy, and redacted of identifiable information, then imported into Dedoose for data management and analysis (Dedoose Version 10.^0^.^35^,^2025^). Initially, three coders (BAK, BH, LJH) chose one transcript each to create an “episode profile” of emergent themes, powerful quotations, and other salient content for the purposes of developing a codebook. Using these themes, the interview guide, and text-entry survey questions, the team developed “parent” and “child” codes using an adapted version of flexible coding (Deterding & Waters, 2021); “parent codes” (e.g., MOAs) were refined during team discussions, and then “child codes” were added as extensions of these parent codes (e.g., MOAs > skills). Each member of the coding team (BAK, BH, LJ, CRK, EQ) collectively coded four interviews to familiarize themselves with the final codebook, then double-coded 26 transcripts in blinded pairs of coders, with each pair specializing in specific parent/child coding, and each coder documenting in memos a rationale for applying codes. During consensus meetings, paired coders and the larger team discussed and resolved discrepancies until coders had minimal to no discrepancies. A further 17 transcripts were single-coded with memos to flag questionable coding for review and resolution with a paired second coder, or the larger team if necessary. An additional five transcripts and all open-ended survey responses were coded solely by the PI (BAK). Finally, we conducted a matrix analysis in Excel to examine themes and to identify bidirectional contributions between MOAs.

Mixed Method Analysis. We examined quantitative and qualitative findings separately, then brought them together through team-based discussions comparing, contrasting, and triangulating findings.

## Results

*Demographics.* Participants (*N* = 65) were mostly (80%) between the ages of 26 and 54 years and self-identified as cisgender females (73%). Over half (53%) identified as heterosexual, and most others identified as gay (20%) or bisexual (15%). A majority identified as Black/African American (65%) or White (34%). Most (92%) were employed full-time, and most completed a 4-year college degree (34%) or a Master’s degree (32%). Participants delivered a range of health services. A majority provided HIV-related services (72%), health education (55%), and/or outreach or peer support (57%). Other common services provided included sexual and reproductive health (34%), public health interventions (26%), supervision, management, or administration of HIV services (25%), and case management (22%). Half of the participants identified their roles as outreach or peer workers (51%), and others were administrators/managers/supervisors (34%), sex educators (28%), or nurses (17%), among others. While a large proportion (42%) had only 1–2 years of experience providing HIV-related health services, others had 5–10 years (22%) and between 10 and 20 years (22%) of experience. Finally, participants reported their client populations included gay/bisexual cisgender men (86%), transgender and gender nonconforming people (79%), heterosexual cisgender women (77%), lesbian/bisexual cisgender women (75%), and heterosexual cisgender men (71%). See Table 1.

**Table 1:** Sample characteristics (N = 65)

Variable	n	(%)	Mean	(SD)
Age (Years)			37.7	(11.4)
Age				
18–25	6	9.2		
26–34	21	32.3		
35–54	31	47.7		
55–64	5	7.7		
65 or over	2	3.1		
Gender identity*				
Cisgender female	46	73.0		
Cisgender male	14	22.2		
Transgender female/transfeminine	3	4.8		
Sexual orientation				
Bisexual	9	15.3		
Gay	12	20.3		
Heterosexual (straight)	31	52.5		
Queer	5	8.5		
Two-spirit	2	3.4		
Unknown	6	9.2		
Race and ethnicity**				
American Indian/Alaskan native	2	3.1		
Asian	2	3.1		
Black or African American	42	64.6		
White	22	33.8		
Other	1	1.5		
Hispanic/Latino	3	4.6		
Employment status				
Employed part-time	3	4.6		
Employed full-time	60	92.3		
Volunteer	2	3.1		
Currently a student	10	15.4		
Highest level of completed education				
High school degree or GED	1	1.5		
Some college	9	13.8		
2-year college degree	5	7.7		
4-year college degree	22	33.8		
Masters degree	21	32.3		
Doctoral degree (Ph.D.)	3	4.6		
Professional degree (JD, M.D.)	3	4.6		
Decline to answer	1	1.5		
Health service(s) delivered**				
Anal health care	8	12.3		
Behavioral health	10	15.4		
Case management	14	21.5		
Health education	36	55.4		
HIV-related services	47	72.3		
Outreach or peer support	37	56.9		
Primary care	6	9.2		
Public health intervention	17	26.2		
Research-related care	11	16.9		
Sexual and reproductive health	22	33.8		
Supervising, managing, administering HIV Services	16	24.6		
Additional services	8	12.3		
Role(s) in health Services**				
Activist or human rights advocate	6	9.2		
Administrator, manager, or supervisor	22	33.8		
Counselor, social worker, or mental health provider	9	13.8		
Medical case manager	6	9.2		
Medical assistant	3	4.6		
Medical doctor	2	3.1		
Nurse	11	16.9		
Outreach or peer worker	33	50.8		
Sex educator	18	27.7		
Additional role in health services	9	13.8		
Population of clients in HIV work**				
Lesbian/bisexual cisgender women	49	75.4		
Gay/bisexual cisgender men	56	86.2		
Transgender and gender nonconforming people	51	78.5		
Heterosexual cisgender men	46	70.8		
Heterosexual cisgender women	50	76.9		
Additional option	6	9.2		
Decline to answer	1	1.5		
Years providing HIV-related health services				
1–2 years	27	41.5		
2–5 years	7	10.8		
5–10 years	14	21.5		
10–20 years	14	21.5		
More than 20 years	1	1.5		
Decline to answer	2	3.1		

^*^n = 63 for Gender Identity; **Not mutually exclusive

Exposure to Strategies. Almost all participants (95.4%) attended the full workshop (Activity 1); only three attended partially. In Region 1, despite encouragement from organizational leadership, midwifery staff in Clinic A declined to participate, with no other declines reported across regions. Many participants (40%) attended coaching calls (Activity 2), with most attending only one out of four sessions. Attendance declined with each additional call, but most (88.5%) participated actively if they joined. All participants were given access to patient-facing visual cues (Activity 3). Of six clinic-specific QI meeting participants in Clinics A and B (Activity 4), three attended all meetings, two attended most, and one attended half. An additional operations staff member at Clinic A with responsibility for electronic intake and medical records systems, though not enrolled in the study, attended all QI meetings in Region 1. Six participants (9.2%) from multiple organizations received ad hoc technical assistance, which covered topics such as the use of patient-facing visual cues within a faith-based organization, development of a client-facing workshop, training-of-trainers, and development of new patient-facing outreach materials.

### Mechanisms of Action: Target Behavior 1

**Fig. 2:**
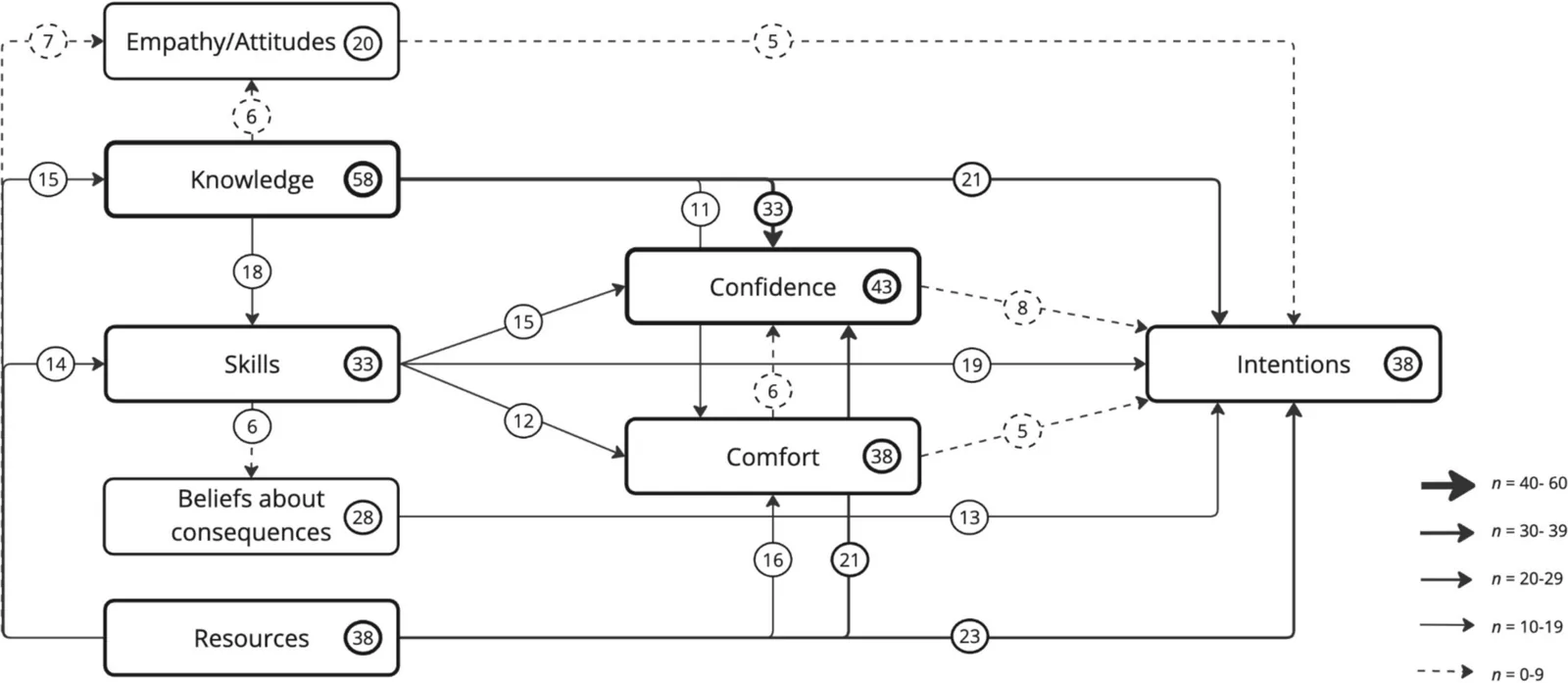
Diagram illustrating the mechanisms of action and their interrelationship based on qualitative analysis

Quantitative findings for MOAs from surveys are summarized in Table 2; detailed item-level findings from Table 2 are in Supplemental Tables 1–3. Qualitative findings are summarized below, with illustrative quotations documented in Table 3. Directional links between MOAs based on qualitative data are depicted in Fig. 2.

**Table 2:** Longitudinal changes in mechanisms of action across implementation periods of PEACHES

	Pre-PEACHES	Post-workshop	Post-PEACHES	Pre-PEACHES v. Post-workshop			Pre- v. Post-PEACHES		
	*Mean* (*SD*)	*Mean* (*SD*)	*Mean* (*SD*)	MD (% MD)	*t* (*df*)	*p*-value	MD (% MD)	*t* (*df*)	*p*-value
Knowledge^†^	51.3 (22.7)	89.5 (10.5)	79.0 (27.8)	38.2 (74%)	14.44 (63)	< .0001	27.7 (54%)	7.05 (64)	< .0001
Skills	2.80 (1.42)	5.19 (0.69)	5.19 (0.76)	2.4 (49%)	12.02 (60)	< .0001	2.4 (48%)	11.88 (55)	< .0001
Positive emotions	4.13 (1.25)	4.92 (0.92)	4.89 (1.02)	0.8 (16%)	5.23 (60)	< .0001	0.7 (14%)	4.36 (54)	< .0001
Negative emotions	2.65 (1.38)	2.06 (1.39)	2.06 (1.34)	− 0.5 (− 11%)	− 2.32 (58)	.0237	− 0.7 (− 14%)	-3.58 (52)	.0007
Professional role and identity	4.70 (0.97)	5.27 (0.79)	5.26 (0.87)	0.6 (12%)	4.34 (62)	< .0001	0.5 (10%)	3.50 (57)	.0009
Informational resources	3.34 (1.41)	5.60 (0.47)	5.51 (0.53)	2.3 (46%)	12.52 (61)	< .0001	2.2 (44%)	11.21 (56)	< .0001
Organizational context and Resources	4.15 (0.99)	4.68 (1.13)	4.73 (1.00)	0.5 (10%)	3.73 (62)	.0004	0.4 (9%)	3.17 (56)	.0025
self-efficacy^††^	3.94 (1.44)	5.14 (0.74)	4.85 (0.88)	1.2 (24%)	6.23 (60)	< .0001	0.9 (19%)	4.80 (55)	< .0001
Ease/confidence Offering Social Support†††	4.03 (0.93)	4.41 (0.52)	4.35 (0.56)	0.37 (9%)	3.35 (63)	.0014	0.32 (8%)	2.87 (58)	.0058
Comfort with anal health assessment (“asking clients about…”)									
sexual orientation	3.45 (0.79)	3.67 (0.56)	3.59 (0.65)	0.22 (5%)	2.50 (63)	.0151	0.14 (3%)	1.59 (57)	.1175
concerns about HIV/STIs	3.54 (0.64)	3.63 (0.60)	3.53 (0.70)	0.08 (2%)	1.22 (63)	.2281	-0.07 (-2%)	-0.66 (58)	.5096
anal sex practices	2.60 (1.20)	3.22 (0.77)	3.25 (0.81)	0.59 (15%)	4.23 (63)	< .0001	0.63 (16%)	4.11 (56)	.0001
concerns about anal sex, apart from HIV/STIs	2.65 (1.28)	3.17 (0.77)	3.33 (0.71)	0.50 (13%)	3.55 (63)	.0007	0.67 (17%)	3.78 (57)	.0004
Comfort with anal health promotion									
Starting a conversation about anal health, apart from HIV/STIs	2.49 (1.26)	3.11 (0.80)	3.14 (0.83)	0.59 (15%)	3.86 (63)	.0003	0.60 (15%)	3.22 (57)	.0021
Responding to concerns about anal health, apart from HIV/STIs	2.46 (1.16)	3.14 (0.75)	3.25 (0.69)	0.67 (17%)	4.77 (63)	< .0001	0.82 (21%)	5.20 (56)	< .0001
Advising how to lessen contact with feces during anal sex	2.17 (1.34)	3.34 (0.78)	3.14 (0.93)	1.14 (29%)	6.85 (63)	< .0001	0.90 (22%)	4.80 (57)	< .0001
Advising how to reduce pain during anal sex ^*††††*^	2.17 (1.39)	3.34 (0.74)	3.24 (0.73)	1.14 (29%)	7.04 (63)	< .0001	1.00 (25%)	5.08 (57)	< .0001
Advising how to make anal sex more pleasurable	2.14 (1.46)	3.20 (0.86)	3.02 (0.95)	1.03 (26%)	6.30 (63)	< .0001	0.81 (20%)	3.93 (57)	.0002
Conducting a sexual history focused on anal sex ^*^	2.42 (1.34)	3.18 (0.80)	3.10 (0.92)	0.73 (18%)	4.63 (55)	< .0001	0.66 (17%)	3.28 (49)	.0019
Collecting anorectal swabs ^*^	3.33 (0.77)	3.67 (0.49)	3.35 (0.79)	0.33 (8%)	1.84 (17)	.0827	0 (0)	0.00 (16)	1
Conducting an anorectal exam ^*††††* *^	2.64 (1.28)	3.29 (0.91)	2.85 (0.99)	0.64 (16%)	1.80 (13)	.0951	0.23 (6%)	0.56 (12)	.5845

Comparisons by timepoint are based only on participants with data at both timepoints; statistical tests compare paired responses and may reflect a reduced sample size. ^†^Significant difference between Post-Workshop and Post-PEACHES driven by three items. See Supplemental Table 1 for statistics for each item

^††^Significant difference between Post-Workshop and Post-PEACHES for Self-efficacy. See Supplemental Table 2 for statistics for each item

^†††^Significant difference between Post-Workshop and Post-PEACHES. See Supplemental Table 3 for statistics for each item

^††††^Significant differences between Post-Workshop and Post-PEACHES for advising on pain (MD =  − 0.17, % MD = -4%, t(57) =  − 2.01, p = .049) and conducting an anorectal exam (MD =  − 0.54, % MD =  − 14%, *t*(12) =  − 2.21, p = .047)

^*^For Comfort items, Pre-PEACHES N = 65, Post-Workshop N = 64, Post-PEACHES N = 59, except last three items were calculated by excluding ‘5 Not part of my role’ across timepoints

**Table 3:** Illustrative quotations for mechanisms of actions from post-workshop and post-PEACHES surveys and interviews among healthcare providers

Mechanisms of action	Illustrative Quotations
Knowledge Awareness of anal pleasure and health acquired as a result of PEACHES	“Knowing some do’s and don'ts, knowing that it doesn’t have to be painful, and it should be pleasurable to the person… a lot of times when you grow up and then you're, especially if you’re growing up a young gay person especially within the Black community, talking about sex, it’s not spoken about. And so, you honestly learn everything on your own…when you don’t know any better, you get information from people who also don’t know any better.”—Participant 2—Post-Workshop “My verbiage with talking to them about certain things, pertaining to the ones that are having anal sex, so just being able to talk a little bit more about what I learned has helped a whole lot. So, that has been a huge help for me, because now I’m not afraid to have those conversations with them.”—Participant 36—Post-PEACHES “I feel like a lot of the times when I spoke to people beforehand, they were from those ‘little t’ truths. From those personal experiences. Now, I feel like I can give them a ‘big T’ truth because it’s pushed by and backed by some hardcore information and hardcore research that I personally didn’t know was out there.”—Participant 58—Post-PEACHES
**Skills** Increased ability or proficiency to perform either Target Behavior 1 or 2 acquired through PEACHES	“So, I can tell you prior to that workshop I didn’t ask somebody, ‘Is your anal sex enjoyable?’ I didn’t really honestly want to know, because I was like my patients share a lot of stuff, and I would just be like no, I don’t want to. But now I feel like when I learned how to do PrEP [pre-exposure prophylaxis for HIV], I feel like I have a lot more knowledge to open up that conversation. Whether they want to have it or not, it’s okay, but at least I feel like I can open it and ask them and be able to share information if I say, ‘Is your sex pleasurable?’”—Participant 55—Post-Workshop “Again, like the ask-offer-ask [from motivational interviewing], asking what they know and then giving them the chance to respond. And then, you know, like asking them would they care to know what I know, and then sharing that with them as well. And then, again, going into the conversation just sharing that anal sex does not have to be painful. It can be 100% painless and pleasurable for those who are engaging. And just letting them know that it’s practiced by a lot of people, just basically destigmatizing anything or like most of the common things that people in our community hear about it before going into their concern.”—Participant 14—Post-Workshop “[After PEACHES,] I like to make sure that people are more comfortable talking about their booty hole and their bottom. It’s definitely – it’s so silly – but one of my rectal swabs that I collect, I like to remind people now that, ‘Hey, this is your anus and your rectum. It’s meant to do a certain thing which is push out poop or fecal matter, and that means when you get this swab for me it might have a little poop on it, and that’s okay.’ And that was not a conversation I was maybe as comfortable having before this thing, which you think as a nurse I would be but it just, that’s another seed I plant. It’s like, ‘Hey, we're going to be comfortable with fact that there's poop on this swab.’”—Participant 33—Post-PEACHES
Empathy/attitudes Understanding and acceptance of the feelings and behaviors of people who engage in anal sex, acquired as a result of PEACHES	“When I look at things, I like to know the science behind everything. And I really enjoyed that we learned the science behind it. Because once you kind of know how everything works together, you understand why other people enjoy anal pleasure and things like that. And you it kind of just takes out all the judgment because you now know the physiology behind everything.”—Participant 4—Post-Workshop “It just made me more knowledgeable of why people enjoy things, whether it’s men, heterosexual men or even women. It just showed me the part why they do it, why they choose to do it because it is pleasure for them. It’s not always pain.”—Participant 11—Post-Workshop
Beliefs about consequences Perceptions about positive results or what will be achieved from undertaking Target Behavior 1 or 2	“I get to bring this information back, not only to my patients, but also to people in my community and friend group, who honestly need this information. I think that they can share that information with their friends and their community, just like I talked about I was going to do with my friends and my husband’s friends. It gets it all out there when it’s needed because let’s face it, you're not talking to your doctor about these things. You're talking to your friends. If one of your friends in the room knows the information, it’s golden. It’s the best thing.”—Participant 33—Post-Workshop “These young men, they feel very defeated. They feel like it’s hopeless. They feel very hopeless, and it’s like, ‘Well, it doesn’t matter, I’m going to get HIV anyway’…They feel like, ‘Well, there’s nothing I can do anyway. I’ll probably just get it’, so they don’t take any kind of precautions. So, if you approach them, I think, from this angle, I think it would be a lot more beneficial."—Participant 8—Post-Workshop “This workshop taught me that if I teach people how to safely engage in anal sex and, like, incorporate the pleasure, like they could naturally prevent themselves from getting certain STIs or STDs, prevent themselves from, like, tearing and hurting themselves. It’s not always about, like, HIV prevention. Like that’s not the main topic. The main topic is, ‘Hey, come and engage in these, or use these tips and tricks to up your pleasure’, and you’re secretly also benefiting from, I guess, like harm reduction-style methods as well.”—Participant 9—Post-Workshop
Informational resources/patient-facing materials changes in a person’s physical or social surroundings resulting from PEACHES that encourage target behavior 1 or 2	“I like the website because it gives the patients ways to engage with their provider as far as, like, feeling comfortable to ask their provider questions? Like before, I think it’s a conversation piece that’s hard for them to talk to their provider. So, now when I give this information to them, especially the person who visited the website, he’s able to come back and have a conversation around anal pleasure. And feels like he’s able to talk about it instead of not saying anything at all. Just coming for the follow-up appointment, he’s able to talk about his issue or questions he might have involving anal sex.”- Participant 27—Post-PEACHES “The palm cards are also a very useful tool to handout to patients. They offer an alternative to providers who might not have the comfort level with anal health but still want to educate their patients.”—Participant 7—Post-PEACHES
Organizational context and resources changes in a person’s physical or social surroundings resulting from PEACHES that encourage target behavior 1 or 2	“I think that the two-day workshop was like an amazing first step. And it definitely got everyone in my practice’s wheels thinking about it. But the whole three and a half months where we’ve had support, working out some of these processes that we started thinking about at the workshop, that’s really solidified for our organization how we do it, why we’re doing it, and the best practices that we can take for these changes.”—Participant 3—Post-PEACHES
Self-Efficacy/ Confidence Increased belief In one’s ability to successfully carry out target behavior 1 or 2 as a result of PEACHES	“It gave me the confidence and the comfortability to be able to kind of talk about these things rather than completely ignoring them and putting up a wall to them, which is what I would have done before when I felt uneducated. Now I feel like I can plant some seeds, and as I work with my patients more when they come back, I can ask these questions and talk to them about these things without a lot of fear and anxiety.”—Participant 33—Post-PEACHES "After the workshop, I realized pleasure and health go hand-in-hand and, in fact, centering pleasure is how we can help people practice harm reduction and engage in safer behaviors. I am now confident that anal pleasure and health can be integrated into settings/environments that are largely conservative, like Alabama.”—Participant 53—Post-Workshop
Comfort and positive & negative emotions feelings of being at ease, comfortable, relaxed with the topic of anal pleasure and health, acquired as a result PEACHES	“I just had some personal stigma around anal pleasure and health, and just maybe living in the part of the country where I do, I was kind of nervous about approaching that subject. So, I think that what, the most powerful thing that the workshop did is it kind of helped with my own personal feelings, allowing me to then be able to broach the subject with clients and not get bogged down by my own maybe personal insecurities or questions or shame or stigma about the subject.”—Participant 3—Post-Workshop “I think feeling more comfortable because beforehand, one, I came in not knowing that much ‘cause I don’t come from like a clinical biological background. So, I think having those resources to interact with, it’s just has built my comfortability and confidence with discussing things like this because even if I get stumbled, I have a resource that I can refer people to. So, I think just me being comfortable in, one, talking about the topic and like I said, my confidence and being able to answer people’s questions and refer them to the information on the website.”—Participant 43—Post-PEACHES
Intentions The conscious decision to perform target behavior 1 or 2 in the future, as a result of PEACHES	“By ourselves or with people that we’re close with, maybe anal sex isn’t a big deal, but one of the points I feel of this workshop is you are now having to talk about something that you might not be used to talking about in a more public setting… which was just a little bit of that social push within my community to say, ‘Anal sex is normal, it’s okay. And look, I’m in this group of other people who work in my city, and we are all learning about it together and addressing it as professionals.’”—Participant 3—Post-Workshop “I definitely think that the study and the client-facing materials all sort of put into perspective that people are having these conversations, maybe not within our walls, but we have to meet people where they are. And if this is what people want, this is what people want. So, I definitely think it showed that we have to meet the mark, so to speak.”—Participant 35—Post-PEACHES “One thing about our organization is we want to be able to, of course, assist our clients in whatever area they may need. I think this brought up an area that we may have missed, per say. But, now that we are trained in being able to have conversations about anal pleasure, it helps make up an area where we may have fallen short.”—Participant 54—Post-PEACHES

#### Knowledge

Before PEACHES, participants answered half (51.3%) of the *iASK* items correctly. After the workshop, their correct answers increased to 89.5% (*p* < .0001, Table 2). Three months later, at the end of PEACHES, participants’ knowledge was still higher than baseline, but 9.3 percentage points lower than Post-Workshop, indicating a significant loss of knowledge over time (*p* < .0027). These drops in knowledge related to douches and enemas, distinctions between the internal and external sphincters, and the function of the pubo-rectal sling muscle (see Supplemental Table 1).

Qualitative data suggest meaningful improvements in knowledge. Fifty-eight participants (89.2%, Fig. 2 and Table 3) reported improved knowledge, particularly in dispelling myths about anal sex and understanding both “layman terms” and “scientific” information about anatomy and physiology. Participants mentioned improved understanding of techniques to relax muscles and reduce pain, the use of lubricants to prevent tearing, and fiber/dietary changes to minimize the risk of contact with feces as well as rectal douche use. Participants noted the value of conveying this knowledge to improve patient engagement in STI/HIV prevention.

#### Skills

Participants’ survey responses indicated a significant 49% increase from Pre-PEACHES to Post-Workshop skills, moving from “Neither disagree nor agree” to “Agree” across statements (*p* < .0001, Table 1). Three months later, this shift was sustained (48%, *p* < .0001), with the largest improvements for starting conversations (76%, improving from “Disagree” to “Strongly agree”) and responding to client questions (60%, improving from “Somewhat disagree” to “Agree”).

Half of the participants reported improved skills qualitatively (Fig. 2 and Table 3), and frequently referred to the Ask-Offer-Ask method from Motivational Interviewing. They found the method highly effective for assessing client knowledge about anal sex, offering information, and increasing client-centered counseling. Another frequently mentioned skill was the pleasure-based approach. Participants appreciated learning to present harm reduction in a pleasure-oriented way, and noticed a “great difference” in their ability to “share information with clients without making it sound weird.” Participants credited their improved skills to changes in comfort and knowledge—specifically, feeling more at ease providing professional information—as well as to the availability of resources to refer clients to during conversations (Fig. 2).

#### Positive and Negative Emotions, Empathy and Attitudes

After the workshop, participants reported feeling significantly more positive emotions (Table 2), specifically, feeling more natural, at ease, and comfortable discussing anal pleasure and health (15–19% increases, all *p* < .0001, Supplemental Table 2). This persisted after PEACHES ended. The scale mean for negative emotions decreased significantly (see Table 2), driven by a 28% drop on a single item: feeling less uncertain about how to discuss anal sex with a client (*p* < .0001, Supplemental Table 2).

Twenty participants (30.8%) remarked in qualitative data (Table 3) that PEACHES helped them to view anal sex as natural and commonplace. They credited this change in empathy and attitude to improvements in their knowledge, skills, and access to credible resources (Fig. 2). They described approaching conversations with less judgment because they had learned a scientific rationale for why people enjoy anal sex, regardless of gender or sexual orientation. Participants also noted that open dialogue about anal health and pleasure in the social environment of PEACHES enabled them to express themselves more directly in public settings.

#### Professional Role and Identity & Beliefs about Consequences

Perception of the professional relevance of addressing anal health and pleasure with clients increased 12% after the workshop (*p* < .0001) and was sustained Post-PEACHES (10%, *p* = .0009, Table 2). At the item level (Supplemental Table 2), participants agreed that their role as HIV workers was to discuss both anal health and anal pleasure (16% and 20% increases, respectively), and they reported feeling responsible for improving their own and their clients’ comfort with these topics (10% and 4% increases, respectively).

In qualitative data, participants emphasized their responsibility to mitigate the impact of anal sex stigma by educating others in accurate, scientifically-guided, compassionate, and nonjudgmental ways. Several (43.1%, Fig. 2 and Table 3) shared that they believed open discussions would dispel myths, reduce injury risks, and increase the acceptability of discussing anal sex, particularly in the South, where sex education is limited. They believed that clients would share information with friends and family, promoting safer, more informed health decisions and comfort within social circles and during healthcare encounters. In particular, communicating about anal pleasure was seen as helping clients feel more in control of their sexual practices and open to lowering their STI and HIV risk.

#### Informational Resources/Patient-Facing Materials

Pre-PEACHES, participants were neutral (*“Neither disagree nor agree”*) about their ability to find, access, and refer clients to “accurate and reliable” information (Supplemental Table 2). Following the workshop, when participants received patient-facing visual cues, the average Likert response increased by 46%, to between *“Agree”* and *“Strongly agree”* (*p* < .0001, Table 2)*.* This improvement was sustained after the intervention (46%, *p* < .0001).

In qualitative data, most participants (58.5%, Table 3) referenced the client-facing materials—including posters, peach- and prostate-shaped squeeze balls, palm cards, and buttons—as great conversation starters, visual reminders, and “game changers” in creating a more open environment for clients. They mentioned that the framed certificate of workshop completion demonstrated expertise and fostered trust with clients by showing that health workers were knowledgeable and equipped to address concerns. Participants most frequently mentioned the website, crediting it for increasing their knowledge, comfort, confidence, and skills in ways that strengthened their intentions to discuss anal pleasure and health (Fig. 2). They believed this informational resource also enabled clients to educate themselves and to be less ashamed about disclosing participation in anal sex.

#### Organizational Context and Resources

Organizational context and resources, referring to participants’ perceptions of their own clinic’s context and resources to promote anal pleasure and health, also shifted significantly by 10% (*p* = .0004) and were sustained after PEACHES ended (9%, *p* = .0025, Table 2). Individual item increases (Supplemental Table 2) varied in significance, with significant Post-PEACHES changes for workplace acceptability (7%, *p* = .0317) and organizational performance (16%, *p* = .0006), but not for leadership support.

In qualitative data, participants noted that their organizations became more open and engaged in promoting the “pleasure principle.” Thirty-eight participants (Fig. 2 and Table 3) reported changes to their physical or social surroundings and resources (e.g., intake forms, standard operating procedures) that encouraged them to address anal pleasure and health in a sex-positive, accurate, and destigmatizing way. QI participants credited meetings for enabling open discussions and changes in intake forms, screenings, and anorectal testing procedures. Coaching sessions increased participants’ motivation to use patient-facing materials and share information with clients.

#### Self-efficacy/Confidence; Ease with Offering Social Support

Perceived ability to discuss anal pleasure and health under challenging circumstances shifted from *“Somewhat agree”* to “*Agree”* (Table 2). While self-efficacy significantly increased overall from Pre- to Post-PEACHES (*p* < .0001), there was a slight drop in confidence (5%, *p* < .0001) between the end of the workshop and the end of PEACHES. In terms of ease/confidence providing informational and emotional social support, there was a significant 9% increase after the workshop, shifting from *“Easy”* to *“Very easy”* (*p* = .0014, Table 2 and Supplemental Table 3)*.* This improvement was maintained Post-PEACHES (8%, *p* = .0058).

Forty-three participants (66.2%, Fig. 2 and Table 3) expressed confidence in their ability to discuss anal pleasure and health with clients without hesitation. Participants credited the shift in confidence to changes in knowledge, resources, and skills (Fig. 2), specifically learning reliable, scientific information about anatomy and physiology, learning how to incorporate dietary supplements to enhance anal pleasure, and learning medical and lay language related to anal sex. Participants reported that the website and other patient-facing materials increased their confidence by serving as a reminder that their information came from a credible source and by providing “many different avenues” to “break the ice” with clients.

#### Comfort with Assessment and Anal Health Promotion

Pre-PEACHES, participants felt “Very” to “Extremely comfortable” asking about clients’ sexual orientation and HIV/STI concerns, and these comfort levels remained stable over time (Table 2). In contrast, their comfort asking about anal sex practices and related concerns apart from HIV/STIs was initially lower (between “Moderately” and “Very comfortable”) and then, after the workshop, increased significantly (15%, *p* < .0001 and 13%, *p* < .0007, respectively) to between “Very” and “Extremely comfortable.” These item responses remained elevated Post-PEACHES as did increases in comfort starting conversations (15%, *p* = .0021), responding to concerns (21%, *p* < .0001), conducting a sexual history (17%, *p* < .0019), and providing advice on reducing fecal contact (22%, *p* < .0001), pain (25%, *p* < .0001), and enhancing pleasure (20%, *p* < .0001). Comfort collecting anorectal swabs and conducting an anorectal exam did not increase among those who reported these activities as part of their role in healthcare.

Qualitative inquiry corroborated the reported increases in comfort (Table 3). Fifty-eight percent credited this to improvements in their knowledge, skills, and resources (Fig. 2). They noted that the scientific information and talking points of PEACHES reduced awkwardness and increased competence to normalize discussion and respond to concerns by eliciting needs and desires across patient populations. Several participants mentioned that access to the visually pleasing website made it easier to broach the topic, since they had a reliable source of information.

#### Intentions (Qualitative Only)

After PEACHES, most participants (58.5%, Fig. 2 and Table 3) reported plans to restructure sexual health assessments and intake processes, integrate workshop materials into their work environments, and engage in conversations about anal pleasure and health with colleagues, family, and friends to help destigmatize and normalize the topic. Participants credited their increased intentions to improvements in knowledge, empathy/attitudes, comfort, confidence, skills, beliefs about consequences, and resources (Fig. 2). They mentioned that acquiring knowledge helped them to be more compassionate and nonjudgmental, and thereby motivated broaching anal pleasure more frequently with their clients. Participants commended the patient-facing materials for helping to normalize the topic and stimulate their intentions to discuss anal pleasure and health in a collaborative way.

**Summary of Target Behavior 1**: Several MOAs, including knowledge, skills, professional identity, and attitudes, shifted during PEACHES. However, resources—whether informational or organization-specific—were the most influential MOAs, precursors to all other MOAs (Fig. 2). Participants commonly credited these patient-facing resources and, for those who instituted organizational changes, clinic-specific QI meetings for boosting knowledge, comfort, skills, and intentions, sustaining improvements, and enabling greater confidence supporting clients and normalizing conversations over time.

### Impact on Behavior (Target Behaviour 2)

#### Frequency of Anal Health Assessment and Promotion

Prior to PEACHES, participants’ survey responses indicated they asked their clients about their anal sex practices and anal sex concerns “Sometimes.” Post-PEACHES (Table 4), participants more frequently asked clients about their anal sex practices (16% increase, *p* = .0002) and anal sex concerns (15% increase, *p* = .0114), moving each closer to “Often.” For anal health promotion, their Post-PEACHES survey responses indicated significant increases, from “Not at all” to “Sometimes,” with the largest shifts related to advising how to lessen contact with feces (20%, *p* < .0001), reducing pain (23%, *p* < .0001), and enhancing pleasure during anal sex (25%, *p* < .0001).

**Table 4:** Longitudinal differences in frequency of assessment and anal health promotion activities in the past 3 months

	Pre-PEACHES	Post-PEACHES				
	*N* = 65	*N* = 58	Pre-PEACHES v. Post-PEACHES			
	*Mean*	*Mean*	*MD*	*% MD*	*t (df)*	*p-value*
Frequency of anal health assessment (“asking clients about their…”)						
sexual orientation	2.43 (1.52)	2.59 (1.27)	0.07	2%	0.40 (57)	.6928
concerns about HIV/STIs	2.85 (1.48)	3.00 (1.23)	-0.05	− 1%	– 0.35 (57)	.7251
anal sex practices	1.09 (1.34)	1.81 (1.16)	0.62	16%	4.05 (57)	.0002
concerns about anal sex, apart from HIV/STIs	1.09 (1.47)	1.78 (1.31)	0.59	15%	2.62 (57)	.0114
Frequency of anal health promotion						
Starting a conversation about anal health, apart from HIV/STIs	0.81 (1.33)	1.64 (1.31)	0.74	19%	4.12 (57)	.0001
Responding to concerns about anal health, apart from HIV/STIs	1.08 (1.50)	1.81 (1.32)	0.62	16%	3.26 (57)	.0019
Advising how to lessen contact with feces during anal sex	0.32 (0.86)	1.16 (1.17)	0.79	20%	5.43 (56)	< .0001
Advised how to reduce pain during anal sex	0.48 (0.99)	1.45 (1.19)	0.91	23%	5.58 (57)	< .0001
Advised how to make anal sex more pleasurable	0.44 (0.97)	1.47 (1.27)	0.98	25%	5.99 (57)	< .0001
Conducting a sexual history focused on anal sex*	0.65 (1.14)	1.45 (1.25)	0.75	19%	4.95 (50)	< .0001
Collecting anorectal swabs*	2.00 (1.62)	2.18 (1.55)	0.18	5%	0.53 (16)	.6053
Conducting an anorectal exam*	1.31 (1.44)	1.62 (1.66)	0.31	8%	1.76 (12)	.1039

Comparisons by timepoint are based only on participants with data at both timepoints; statistical tests compare paired responses and may reflect a reduced sample size. All item Likert responses ranged from 0 to 4, with higher scores indicating greater frequency: 0 Not at all, 1 Sometimes, 2 Often, 3 Very Often, 4 Always; % MD = MD/(Scale Range [4]). * Calculated by excluding “5 Not part of my role” across timepoints

Qualitative findings on the frequency of anal health promotion are summarized below in terms of the following themes.

#### Passive Dissemination

Twenty-five participants (38.5%) reported passively disseminating patient-facing materials. For instance, they placed magnets, stickers, palm cards, and peach- and prostate-shaped squeeze stress balls throughout the clinic for people to grab, and put up posters and certificates of workshop completion on their walls. Clinic A (Region 1) reported purchasing frames for their posters so they would last longer. This passive dissemination stimulated impromptu conversation and also made actively broaching the topic easier.

“The button. I have it up in my office. When I’m on a Zoom call or something, it’s right in the background so people can see it… I have had some people in the office come around and ask me about it. So, it does bring attention and make people open to talk about anal sex and anal pleasure.”—Participant 17 (Post-PEACHES).

“We always ask about anal sex, but we never really asked about, ‘How do you as the patient feel about anal pleasure? Or is that something that you want to discuss?’ Now that we have the right cards in the room, it’s easier to bring up the conversation.”—Participant 27 (Post-PEACHES).

“The ‘butt’ (peaches) stress balls, the pins and stickers were fun especially to just stick around the city or wherever I travelled just to spread general awareness to let people know they are not alone!”—Participant 24 (Post-PEACHES).

#### Active Dissemination

Thirty-five (53.8%) participants reported actively handing out pins, magnets, palm cards, stickers, and peach- and prostate-shaped stress balls at outreach events; directing people to the workshop website; and actively discussing anal pleasure and health with clients and colleagues.

#### Program Planning

Fourteen (21.5%) participants reported incorporating anal pleasure and health into existing or new programming at their organizations. Participants described reevaluating organizational protocols and client screening processes and updating intake forms (adding questions to promote more discussion about anal pleasure and health), and improving access to anorectal STI screening. After attending an ad hoc technical assistance meeting with the PEACHES team, two participants created a workshop for clients using content and activities from the health worker workshop.

#### Personal Reports

Thirteen participants (20.0%) shared personal accounts of how PEACHES benefited sexual health for clients, colleagues, and friends as well as themselves.

“Even when I engage in sex, I’m more aware of the communication coming from the partner that I’m with if things are painful, if things are pleasurable. ‘It’s not supposed to bleed,’ like those things are really simple, but they are things, like you said, in the workshop that we just think as a society are supposed to happen because we’re dealing with such a sensitive area.” Participant 22 (Post-Workshop).

Participants reported using anatomical knowledge and techniques, such as muscle relaxation and breathing exercises, to reduce strain. Several also reported starting fiber supplements, experimenting with different types of lubricants, and researching different ways to clean their rectums.

### Uptake of HIV-Related Services

Aggregated monthly data (Table 5) signaled that PEACHES had an impact on patient uptake of anorectal STI screening in Clinic A: testing rates increased from 24.2% of STI patients before PEACHES to 38.7% during PEACHES—a 60% relative increase—and remained elevated at 36.8% three months post-PEACHES, representing a sustained 52% increase from baseline. There was also a 28% increase in new PrEP prescriptions, but no other clear impact at the clinic level. At Clinic B, anorectal testing declined over the study period and follow-up, with no notable improvements in other HIV-related outcomes.

**Table 5:** Uptake of HIV-related services based on aggregated monthly health record data at two clinics that received organization-specific quality improvement meetings

	Pre-PEACHES 9/1/2023—11/30/2023	During PEACHES 12/1/2023—2/29/2024	Post-PEACHES 3/1/2024—5/30/2024
*Region 1 Clinic A (Tennessee)*			
STI Screening among Individual Patients*			
Anorectal Samples/Total STI Samples	8/33 (24.2%)	12/31 (38.7%)	20/55 (36.4%)
Oropharyngeal Samples/Total STI Samples	30/33 (90.9%)	30/31 (96.8%)	50/55 (90.9%)
Urethral Samples/Total STI Samples	23/33 (69.7%)	26/31 (83.9%)	43/55 (78.2%)
New PrEP Prescriptions/Total STI Samples	5/33 (15.2%)	6/31 (19.4%)	3/55 (5.5%)
HIV Tests Ordered/Total Clinic Visits	52/500 (10.4%)	59/467 (12.6%)	66/687 (9.6%)
*Region 2 Clinic B (Mississippi)*	Pre-PEACHES 3/1/2024—5/30/2024	During PEACHES 6/1/2024—8/31/2024	Post-PEACHES 9/1/2024—11/30/2024
STI Screening among Patients			
Anorectal Patients/Total STI Patients	92/182 (50.5%)	94/224 (42.0%)	75/204 (36.8%)
Oropharyngeal Patients/Total STI Patients	157/182 (86.3%)	180/224 (80.4%)	171/204 (83.8%)
Urethral Patients/Total STI Patients	165/182 (90.7%)	200/224 (89.3%)	191/204 (93.6%)
New PrEP Prescriptions/Total non-HIV Care Visits	15/116 (12.9%)	17/160 (10.6%)	8/161 (5.0%)
HIV Tests/Total non-HIV Care Visits	100/116 (86.2%)	151/160 (94.4%)	132/161 (82.0%)

^*^Individual patients may be counted more than once across anorectal, oropharyngeal, urethral gonorrhea and Chlamydia screening; some individual patients may have repeated screening within the same period of time. On April 1, 2024, Title X funding arrived and allowed the clinic to pay for three-site testing unrelated to the study intervention

## Discussion

We evaluated the impact of PEACHES, a package of anti-stigma training and quality improvement strategies to enable HIV workers to address anal sex and health in a holistic, pleasure-inclusive fashion. PEACHES was designed to mitigate stigma across multiple socio-ecological levels (Cook et al., 2014; Richman & Hatzenbuehler, 2014). We targeted individual HCW changes with group-level training while introducing clinic-level changes through patient-facing visual cues and quality improvement meetings. Our findings showed significant and sustained positive impact on mechanisms of action for communication about and promotion of anal pleasure and health, and a signal of improvements in anorectal STI screening at one out of two clinics where medical record data was assessed.

PEACHES sought to mitigate anal sex stigma by providing accurate physiological information about anal sex, teaching communication skills, and encouraging environmental changes within clinical settings—all to normalize conversations about anal pleasure and health as a relevant topic for discussion within HIV services. In our study, comfort assessing anal sex practices and concerns apart from HIV/STIs rose significantly, as did comfort with anal health promotion and other key mechanisms of action like knowledge, skills, and self-efficacy. Previous research suggests healthcare workers’ lack of knowledge and fear of communicating inaccurate information hinder their ability to provide adequate care to clients (Zarei et al., 2015) and exacerbate negative attitudes (Dunbar et al., 2020), while effective communication and positive client engagement encourage treatment utilization (Spence et al., 2022). In addition to group-level training, participants described how QI strategies such as the placement of patient-facing visual cues linked to a website of informational resources and organization-specific changes to anal sex-related health services strongly influenced all other mechanisms of action for discussing anal pleasure and health. Increasingly, implementation strategies have incorporated community-level and institutional-level approaches to affect changes in individual-level stigma mechanisms (Rao et al., 2019), as single-level strategies are insufficient to address the multi-layered nature of stigma but may work well in tandem with broader intrapersonal and structural strategies (Cook et al., 2014; Heijnders & Meij, 2006). We found that employing multi-level strategies was pivotal to promoting holistic care. Specifically, participants causally linked their intentions to address anal pleasure and health to the availability of resources (Fig. 2).

Data from two clinics that received organization-specific QI meetings revealed diverging impact. Clinic A increased anorectal STI testing rates and new PrEP prescriptions, while oropharyngeal and urethral screening rates remained relatively stable. This supports a potential intervention effect. At Clinic B, anorectal testing and PrEP prescriptions declined over time. Population fluctuations in this clinic’s college town may partially explain this decline, and suggest the need for 12-month observation periods, rather than our pilot study’s 3-month periods, to remove seasonal effects (Zhang et al., 2020). However, additional factors likely contributed to this decrease because Clinic B’s oropharyngeal and urethral STI screening rates remained stable during this time, and overall STI testing volume rose. These divergent findings should be interpreted cautiously but point to the potential value in examining implementation processes at each clinic, already underway for our study, to understand contextual drivers of these diverging results.

Our study’s design and potential for bias limit our findings in several ways. Pre/post-designs are not equipped to ascertain causality, and our sample size precludes accounting for potential confounds, such as participants’ sexual orientation and their own participation in anal sex. Additionally, selection bias could limit the generalizability of our findings, as those who are willing to learn about anal pleasure and health may harbor less stigmatizing attitudes than the general population of HIV workers. Relatedly, even though nearly all ratings on mechanisms of action improved over time, some initial scores, like ease/confidence offering informational and emotional social support, were already moderate to high. Self-report measures capture perceptions, not actual skill, and can be inflated by limited self-awareness prior to exposure to behavior change techniques, as suggested by the Dunning-Kruger effect (Kruger & Dunning, 1999). A retrospective pre-post-assessment would enable participants to assess their abilities after exposure to PEACHES, accounting for this potential bias. Finally, reporting bias may have skewed our results, as the purveyor of PEACHES (BAK) led the research team that elicited and analyzed its assessment, potentially influencing participants and the research team toward more positive reporting and interpretation.

Future research should evaluate PEACHES for causality and sustained impact with longer observation periods for client outcomes, controlling for time and nesting within regions and organizations, differential exposure to strategies across clinics and participants, as well as other potential confounds in a fully-powered hybrid implementation-effectiveness trial. Given the multi-level nature of PEACHES, HCWs who did not attend the workshop, coaching calls or QI sessions, yet who worked within clinics that changed their organizational practices, may also have been impacted by social and environmental changes within participants and clinics; future studies could further explore this. Additionally, future studies could examine the inclusion of patients, as this could allow them to take an active role and motivate sustained efforts at stigma reduction within organizations (Andersson et al., 2019; Heijnders & Meij, 2006). Additionally, future stigma studies should consider robust evaluation of implementation processes. Although PEACHES demonstrated significant improvements in key mechanisms of action across participants, divergent clinic-level outcomes in anorectal SIT testing and PrEP prescription underscore the importance of attending to contextual factors and organizational implementation conditions that potentially shape how HCWs operate in their clinical settings. Our findings link with previous calls to emphasize attention to implementation science, especially in the field of stigma intervention, where variability in implementation fidelity may substantially influence downstream clinical outcomes (Kemp et al., 2019; Mustanski et al., 2024).

Our findings suggest several implications for healthcare policy and practice. First, even among HIV health workers who were already professionally supported or identified as sexual and gender minorities, significant changes in mechanisms of action were still observed. This strongly suggests that an already sensitized HIV workforce can still benefit from training and quality improvement related to anal sex, and aligns with findings that anal sex stigma intersects with sexual and gender minority stigma as well as HIV stigma, while also being sufficiently distinct to warrant specific intervention (Dangerfield & Turpin, 2025; Kutner et al., 2020a, 2020b, 2020c, 2020d). Second, our results highlight the value of structural interventions, such as inclusive policies, institutional practices, active community engagement, and environmental and material resources—reflected in our QI strategies—in enhancing the effectiveness of stigma-reduction efforts and improving health outcomes. These structural supports may create clinical environments that normalize conversations about anal sex and reduce barriers to disclosure, thereby improving patient experiences and health outcomes (Hatzenbuehler et al., 2013; Stangl et al., 2019). Third, our results highlight the potential benefit of real-time data monitoring. Clinics A and B did not see their patient-level outcomes until after PEACHES ended, but real-time data monitoring of patient-level data with feedback at the provider-level could help clinics track trends as they occur and motivate additional site-specific improvements to service delivery (Ivers et al., 2012). This monitoring could employ inferential statistics, something our pilot study’s crude comparison of aggregated clinic-level outcomes could not conduct. Lastly, future efforts should identify and support HIV workers who are willing to learn about anal pleasure and health as local champions within their organizational contexts. Empowering willing providers to model inclusive practices and advocate for institutional changes could catalyze organizational changes that increase patient comfort to discuss anal sex and health, and ultimately improve provider adoption and patient uptake of anal sex-related HIV interventions like anorectal STI screening and PrEP.

## Supplementary Information

Below is the link to the electronic supplementary material.

Supplementary file1 (DOCX 45 kb)

## Data Availability

Data requests will be reviewed upon request of the corresponding author.
