# Supplementary material for: Evaluating PEACHES: Pleasure-Inclusive Implementation Strategies to Enable Healthcare Workers to Address Anal Sex Stigma During HIV Service Encounters
Source: Arch Sex Behav. 2026 Aug 19;55(6):2701–21. doi: 10.1007/s10508-026-03500-7 (PMC13597607; doi:10.1007/s10508-026-03500-7)
Supplement: Supplementary file 1 [file 10508_2026_3500_MOESM1_ESM.docx]

# Supplemental File 1. Detailed specification of activities within the PEACHES package of implementation strategies to enable addressing anal sex stigma among 65 HIV workers in Tennessee, Alabama and Mississippi

| Strategies | **HCW-level Training** | | **Clinic-level Quality Improvement** | |
| --- | --- | --- | --- | --- |
| Components of Strategies | Workshop | Coaching Calls | Placement of Patient-Facing Visual Cues | Tailored Quality Improvement Sessions (only in Clinic A and Clinic B) |
| Description | A 2-day in-person “Smarter Sex Workshop” | Four hour-long coaching sessions | Placement of pins, posters, and other visual materials with anti-stigma messages and a QR/URL for a website of supportive resources | Four hour-long QI session for site-specific support to develop and implement organization-specific, clinic-level environmental restructuring |
| Actor delivering the strategy | Principal Investigator (BAK) | Principal Investigator (BAK) | Principal Investigator (BAK) | Principal Investigator (BAK), with intermittent support from an SE AETC practice transformation coach |
| Action | Values clarification: stigma toward anal sex, professional role & identity  Interactive didactics: anal sex prevalence, anatomy and physiology, sexual response; information exchange strategies from Motivational Interviewing  Role plays: case scenarios based on common patient questions | Skills-practice sessions and opportunity for tailored feedback and guidance to maintain and reinforce knowledge and skills from the workshop | Provision of visual cues for introduction into clinic environments and outreach events | Clinic A: Iteration of patient-facing poster; consultation on placement of visual cues; revision of electronic intake and return visit assessment forms to reduce stigmatizing language and include anal pleasure and health; draft revisions for electronic health record to include anal pleasure and health; clinic flow for anorectal STI screening and development of a new standard operating procedure (SOP)  Clinic B: Revision of intake and return visit assessment forms to reduce stigmatizing language and include anal pleasure and health; consultation on placement of visual cues  For both Clinic A and Clinic B, additional opportunities to confer via email and phone as needed |
| Action target | Healthcare workers with responsibility for the delivery of HIV-related services | Healthcare workers with responsibility for the delivery of HIV-related services | Status-neutral patient population accessing services at each clinic | Site staff from Clinic A and Clinic B who could adopt and organizational leadership who could support anal health service delivery |
| Temporality (strategy sequence) | First step in delivery of the PEACHES package of strategies | Within one month of workshop delivery | Delivery of materials to participants immediately after the workshop | Within one month of workshop delivery |
| Dose | One workshop (16 hours), over 2 consecutive days | Four 60-minute virtual sessions, once every 3-4 weeks over the course of 3 months | Distributed/placed in clinics and outreach events within one month of the workshop, with expanded delivery of additional materials upon request | Four 60-minute virtual sessions per clinic, once every 3-4 weeks over the course of 3 months |
| Outcomes | Target Behavior 1: Mechanism of Action: Comfort with assessment and anal health promotion activities, Knowledge, Confidence offering informational and emotional social support, Skills, Empathy and Attitudes, Beliefs about Consequences, Organizational Context & Resources, Positive and Negative Emotions; Informational Resources, and Intentions (see Tables 2 and 3, Supplemental Tables 1 through 3, Figure 2)  Target Behavior 2: Impact on Behavior: Frequency of assessment and anal health promotion activities (Table 5); Passive dissemination, Active dissemination, Program Planning, Personal Reports (see Results); Service outcomes of patient uptake of anal sex-related HIV services (see Tables 6 and 7) | | | |
| Theoretical Justification | Training provides *skills*, *knowledge* and modeling (COM-B Model); cognitive reframing and gradual exposure to information and sensitive topics, with information exchange *skills* (Motivational Interviewing), to increase HCW *comfort* & *confidence* | Social learning theory, that people learn best from modelling of others, consistent with COM-B Model that posits that social opportunity (modeling) enhances capability (*skills*, *knowledge*) and motivation (*confidence*) | COM-B Model posits changing the physical environment (opportunity) can change automatic motivation (reducing *emotional discomfort*) to normalize services within the clinic by signalling acceptance and reducing anticipated stigma among patients | COM-B Model posits that enhancing psychological capability among clinic staff and enabling opportunity to examine and alter clinic-level barriers to care should improve the quality of care and patient uptake of anal sex-related HIV services |

# Supplemental File 2: Categorization of each PEACHES implementation strategy activity in terms of Intervention Types (ITs) and Behavior Change Techniques (BCTs) based on the Behaviour Change Wheel and the Behaviour Change Intervention Ontology

See [link](https://docs.google.com/spreadsheets/d/1zvgC8cyu5s0QwTV9o3NU_bdxBzLwHjTRopwjRXQvkqs/edit?usp=sharing)

|  | | **PEACHES Anti-Stigma Implementation Strategies** | | | |  |  |
| --- | --- | --- | --- | --- | --- | --- | --- |
|  |  | **Training Strategies** | | **Quality Improvement Strategies** | |  |  |
| **Intervention Types (ITs) from the**  **Behaviour Change Wheel (BCW)** | | Activity 1:  Workshop | Activity 2:  Coaching Calls | Activity 3:  Patient-Facing Visual Materials | Activity 4:  QI Meetings (Clinic A & B) | Activities with IT | % of Activities with IT |
| 1. Education | | ✓ | ✓ | ✓ | ✓ | 4 | 100% |
| 2. Persuasion | | ✓ | ✓ | ✓ | ✓ | 4 | 100% |
| 5. Training | | ✓ | ✓ |  |  | 2 | 50% |
| 7. Environmental restructuring | | ✓ | ✓ | ✓ | ✓ | 4 | 100% |
| 8. Enablement | | ✓ | ✓ |  | ✓ | 3 | 75% |
| 9. Modeling | | ✓ | ✓ |  |  | 2 | 50% |
| Frequency of ITs in each Activity | | 6 | 6 | 3 | 4 |  |  |

|  |  | **PEACHES Anti-Stigma Implementation Strategies** | | | |  |  |
| --- | --- | --- | --- | --- | --- | --- | --- |
|  |  | **Training Strategies** | | **Quality Improvement Strategies** | |  |  |
| **Behaviour Change Techniques (BCTs) from the BCTT v1.0** | Hyperlink to Behaviour Change Intervention Ontology (BCIO) | Activity 1: Workshop | Activity 2: Coaching Calls | Activity 3: Patient-Facing Visual Materials | Activity 4: QI Meetings (Clinic A & B) | Activities with BCT | % of Activities with BCT |
| 1.1 Goal setting (behavior) | [BCIO_007003](https://bciosearch.org/BCIO_007003) | ✓ | ✓ |  | ✓ | 3 | 60% |
| 1.2 Problem solving | [BCIO_007008](https://www.bciosearch.org/BCIO_007008) |  | ✓ |  | ✓ | 2 | 40% |
| 1.3 Goal setting (outcome) | [BCIO_007005](https://www.bciosearch.org/BCIO_007005) |  |  |  | ✓ | 1 | 20% |
| 1.4 Action planning | [BCIO_007010](https://www.bciosearch.org/BCIO_007010) |  | ✓ |  | ✓ | 2 | 40% |
| 1.5 Review behavior goal(s) | [BCIO_007011](https://www.bciosearch.org/BCIO_007011) |  | ✓ |  | ✓ | 2 | 40% |
| 1.6 Discrepancy between current behavior and goal | [BCIO:007012](https://bciosearch.org/BCIO_007012) | ✓ | ✓ |  | ✓ | 3 | 60% |
| 1.7 Review outcome goals | [BCIO_007013](https://www.bciosearch.org/BCIO_007013) |  |  |  | ✓ | 1 | 20% |
| 1.8 Behavioral contract | [BCIO_007014](https://www.bciosearch.org/BCIO_007014) |  |  |  | ✓ | 1 | 20% |
| 1.9 Commitment | [BCIO_007015](https://bciosearch.org/BCIO_007015) | ✓ | ✓ |  | ✓ | 3 | 60% |
| 2.1 Monitoring of behavior by others without feedback | [BCIO_007021](https://www.bciosearch.org/BCIO_007021) | ✓ |  |  | ✓ | 2 | 40% |
| 2.2 Feedback on behavior | [BCIO_007022](https://www.bciosearch.org/BCIO_007022) | ✓ | ✓ |  | ✓ | 3 | 60% |
| 2.5 Monitoring outcomes of behavior by others without feedback | [BCIO_007020](https://www.bciosearch.org/BCIO_007020) |  | ✓ |  | ✓ | 2 | 40% |
| 2.7 Feedback on outcome(s) of behavior | [BCIO_007027](https://www.bciosearch.org/BCIO_007027) |  |  |  | ✓ | 1 | 20% |
| 3.1 Social support (unspecified) | [BCIO_050397](https://www.bciosearch.org/BCIO_050397) | ✓ | ✓ |  |  | 3 | 60% |
| 3.2 Social support (practical) | [BCIO_007028](https://www.bciosearch.org/BCIO_007028) | ✓ | ✓ |  |  | 3 | 60% |
| 3.3 Social support (emotional) | [BCIO_007041](https://www.bciosearch.org/BCIO_007041) |  | ✓ |  |  | 2 | 40% |
| 4.1 Instruction on how to perform a behavior | [BCIO_007058](https://www.bciosearch.org/BCIO_007058) | ✓ | ✓ |  | ✓ | 3 | 60% |
| 4.2 Information about antecedents | [BCIO_007052](https://www.bciosearch.org/BCIO_007052) |  |  |  | ✓ | 1 | 20% |
| 4.3 Re-attribution | [BCIO_007053](https://www.bciosearch.org/BCIO_007053) |  | ✓ |  |  | 2 | 40% |
| 4.4 Behavioral experiments | [BCIO_007054](https://www.bciosearch.org/BCIO_007054) |  |  |  | ✓ | 1 | 20% |
| 5.1 Information about health consequences | [BCIO_007063](https://www.bciosearch.org/BCIO_007063) | ✓ | ✓ |  |  | 3 | 60% |
| 5.2 Salience of consequences | [BCIO_007068](https://www.bciosearch.org/BCIO_007068) | ✓ | ✓ |  | ✓ | 3 | 60% |
| 5.3 Information about social and environmental consequences | [BCIO_007064](https://bciosearch.org/BCIO_007176)  [BCIO_007176](https://bciosearch.org/BCIO_007176) | ✓ |  |  |  | 2 | 40% |
| 5.4 Monitoring of emotional consequences | [BCIO_007066](https://www.bciosearch.org/BCIO_007066) | ✓ |  |  |  | 2 | 40% |
| 6.1 Demonstration of the behavior | [BCIO_007055](https://www.bciosearch.org/BCIO_007055) | ✓ | ✓ |  |  | 3 | 60% |
| 6.2  Social comparison | [BCIO_007073](https://www.bciosearch.org/BCIO_007073) | ✓ | ✓ |  |  | 3 | 60% |
| 6.3 Information about others' approval | [BCIO_007074](https://www.bciosearch.org/BCIO_007074) | ✓ | ✓ |  |  | 3 | 60% |
| 7.1 Prompts/cues | [BCIO_007239](https://www.bciosearch.org/BCIO_007239) | ✓ | ✓ | ✓ |  | 3 | 60% |
| 7.5 Remove aversive stimulus | [BCIO_050331](https://www.bciosearch.org/BCIO_050331) |  |  |  | ✓ | 1 | 20% |
| 7.6 Satiation | [BCIO_007087](https://www.bciosearch.org/BCIO_007087) |  |  |  | ✓ | 1 | 20% |
| 7.7 Exposure | [BCIO_007085](https://www.bciosearch.org/BCIO_007085) | ✓ | ✓ |  | ✓ | 3 | 60% |
| 7.8 Associative learning | [BCIO_007090](https://www.bciosearch.org/BCIO_007090) |  |  |  | ✓ | 1 | 20% |
| 8.1 Behavioral practice/ rehearsal | [BCIO_007094](https://www.bciosearch.org/BCIO_007094) | ✓ | ✓ |  | ✓ | 3 | 60% |
| 8.2 Behavior substitution | [BCIO_050846](https://www.bciosearch.org/BCIO_050846) |  | ✓ |  |  | 2 | 40% |
| 8.3 Habit formation | [BCIO_007096](https://www.bciosearch.org/BCIO_007096) |  | ✓ |  |  | 2 | 40% |
| 8.4 Habit reversal | [BCIO_007097](https://www.bciosearch.org/BCIO_007097) |  |  |  | ✓ | 1 | 20% |
| 8.7 Graded tasks | [BCIO_007100](https://www.bciosearch.org/BCIO_007100) | ✓ | ✓ |  |  | 3 | 60% |
| 9.1 Credible source | [BCIO_007075](https://www.bciosearch.org/BCIO_007075) | ✓ | ✓ | ✓ | ✓ | 3 | 60% |
| 9.3 Comparative imagining of future outcomes | [BCIO_007070](https://www.bciosearch.org/BCIO_007070) | ✓ | ✓ |  |  | 3 | 60% |
| 10.3 Non-specific reward | [BCIO_007253](https://www.bciosearch.org/BCIO_007253) |  | ✓ |  | ✓ | 2 | 40% |
| 10.4 Social reward | [BCIO_007265](https://www.bciosearch.org/BCIO_007265) | ✓ | ✓ |  |  | 3 | 60% |
| 10.5 Social incentive | [BCIO_007218](https://www.bciosearch.org/BCIO_007218) |  |  |  | ✓ | 1 | 20% |
| 10.6 Non-specific incentive | [BCIO_007205](https://www.bciosearch.org/BCIO_007205) |  |  |  | ✓ | 1 | 20% |
| 10.10 Reward (outcome) | [BCIO_007264](https://www.bciosearch.org/BCIO_007264) |  |  |  | ✓ | 1 | 20% |
| 12.1 Restructuring the physical environment | [BCIO_050348](https://www.bciosearch.org/BCIO_050348) | ✓ | ✓ | ✓ | ✓ | 3 | 60% |
| 12.2 Restructuring the social environment | [BCIO_050349](https://www.bciosearch.org/BCIO_050349) | ✓ | ✓ |  |  | 3 | 60% |
| 12.5 Adding objects to the environment | [BCIO_007156](https://www.bciosearch.org/BCIO_007156) | ✓ | ✓ | ✓ | ✓ | 3 | 60% |
| 13.1 Identification of self as role model | [BCIO_007158](https://www.bciosearch.org/BCIO_007158) | ✓ | ✓ |  |  | 3 | 60% |
| 13.2 Framing/ reframing | [BCIO_007302](https://www.bciosearch.org/BCIO_007302) | ✓ | ✓ |  |  | 3 | 60% |
| 13.4 Valued self-identity | [BCIO_007159](https://www.bciosearch.org/BCIO_007159) | ✓ |  |  |  | 2 | 40% |
| 13.5 Identity associated with changed behavior | [BCIO_007160](https://www.bciosearch.org/BCIO_007160) | ✓ |  |  |  | 2 | 40% |
| 14.4 Reward approximation | [BCIO_007253](https://www.bciosearch.org/BCIO_007253) |  | ✓ |  |  | 2 | 40% |
| 15.1 Verbal persuasion about capability | [BCIO_007137](https://www.bciosearch.org/BCIO_007137) |  | ✓ |  |  | 2 | 40% |
| 15.3 Focus on past success | [BCIO_007139](https://www.bciosearch.org/BCIO_007139) |  | ✓ |  |  | 2 | 40% |
| 15.4 Self-talk | [BCIO_007140](https://www.bciosearch.org/BCIO_007140) | ✓ |  |  |  | 2 | 40% |
| 16.2 Imaginary reward | [BCIO_007119](https://www.bciosearch.org/BCIO_007119) | ✓ |  |  |  | 2 | 40% |
| 16.2 Vicarious consequences | [BCIO_007120](https://www.bciosearch.org/BCIO_007120)  [BCIO_007121](https://bciosearch.org/BCIO_007121) |  | ✓ |  |  | 2 | 40% |
| Frequency of BCTs in Each Activity | | 31 | 37 | 4 | 30 |  |  |

# Supplemental Table 1. *Longitudinal Changes across Implementation Periods for Knowledge based on the Inventory of Anal Sex Knowledge (iASK)*

|  | Pre-PEACHES |  | Post-Workshop |  | Post-PEACHES |  | Pre-PEACHES v. Post-Workshop | | |  | Pre- v. Post-PEACHES | | |
| --- | --- | --- | --- | --- | --- | --- | --- | --- | --- | --- | --- | --- | --- |
|  | *Mean Score* (*SD*) |  | *Mean Score* (*SD*) |  | *Mean Score* (*SD*) |  | *MD* | *t* (*df*) | *p*-value |  | *MD* | *t* (*df*) | p-value |
|  | 51.3 (22.7) |  | 89.5 (10.5) |  | 79.0 (27.8) |  | 38.2 | 14.44 (63) | < .0001 |  | 27.7 | 7.05 (64) | < .0001 |
|  | *% Correct* |  | *% Correct* |  | *% Correct* |  | % Diff | ᙭² (*df*) | *p*-value |  | % Diff | ᙭² (*df*) | p-value |
| Douches and enemas should use only warm water.^†^ | 49.2 |  | 89.1 |  | 74.6 |  | 40.6 | 20.83 (63) | <.0001 |  | 25.4 | 7.26 (58) | .0071 |
| A fiber-rich diet naturally clears poop from the rectum. | 86.2 |  | 98.4 |  | 98.3 |  | 12.5 | 4.90 (63) | .0269 |  | 13.6 | 4.90 (58) | .0269 |
| The rectum makes enough lubrication for anal penetration. | 84.6 |  | 89.1 |  | 89.8 |  | 4.7 | 0.31 (63) | .5791 |  | 3.4 | 0.10 (58) | .7518 |
| The internal anal sphincter relaxes open more easily than the external anal sphincter.^†^ | 18.5 |  | 65.6 |  | 42.4 |  | 46.9 | 24.74 (63) | <.0001 |  | 23.7 | 7.04 (58) | .0080 |
| The prostate can be felt two inches inside the rectum, by pressing toward the belly button. | 50.8 |  | 90.6 |  | 98.3 |  | 40.6 | 22.32 (63) | <.0001 |  | 47.5 | 24.30 (58) | <.0001 |
| Pressure on the prostate is pleasurable. | 58.5 |  | 89.1 |  | 89.8 |  | 29.7 | 14.09 (63) | .0002 |  | 30.5 | 13.14 (58) | .0003 |
| Rubbing inside the rectum massages the internal base of the penis. | 29.2 |  | 73.4 |  | 78.0 |  | 43.8 | 20.25 (63) | <.0001 |  | 47.5 | 22.78 (58) | <.0001 |
| The only way to tell if a person with a penis enjoys anal stimulation is if their penis is erect. | 60.0 |  | 96.9 |  | 91.5 |  | 37.5 | 22.04 (63) | <.0001 |  | 32.2 | 14.09 (58) | .0002 |
| During anal stimulation, the natural feeling of having to pee or poop reduces with relaxation. | 49.2 |  | 82.8 |  | 89.8 |  | 32.8 | 14.81 (63) | .0001 |  | 39.0 | 17.93 (58) | <.0001 |
| It is possible to have anal sex with zero physical discomfort. | 53.8 |  | 92.2 |  | 96.6 |  | 37.5 | 18.89 (63) | <.0001 |  | 40.7 | 22.04 (58) | <.0001 |
| The legs of the clitoris explain why some people who have a vagina achieve orgasm during anal penetration. | 27.7 |  | 98.4 |  | 94.9 |  | 70.3 | 41.19 (63) | <.0001 |  | 66.1 | 35.22 (58) | <.0001 |
| Pelvic floor muscles can be exercised to enhance orgasm and control of anal muscles. | 66.2 |  | 96.9 |  | 96.6 |  | 31.2 | 16.41 (63) | <.0001 |  | 30.5 | 13.14 (58) | .0003 |
| The pubo-rectal sling muscle is responsible for 80% of continence and the first curve in the rectum.^†^ | 24.6 |  | 93.8 |  | 84.7 |  | 68.8 | 40.20 (63) | <.0001 |  | 59.3 | 33.03 (58) | <.0001 |
| The presence of blood indicates damage to the anus or rectum. | 60.0 |  | 96.9 |  | 93.2 |  | 37.5 | 22.04 (63) | <.0001 |  | 32.2 | 17.05 (58) | <.0001 |

*Note. Comparisons by timepoint are based only on participants with data at both timepoints; statistical tests compare paired responses and may reflect a reduced sample size.*

# ^†^*Significant difference between Post-Workshop and Post-PEACHES (% Diff = -9.3%, t(63) = -3.13, p = .0027), driven by these items (in order of appearance): % Diff = -15.3%, t(58) = 4.92, p = .0265; % Diff = -22.0%, t(58) = 5.76, p = .0164; % Diff = -11.9%, t(58) = 4.00, p = .0455.*

# Supplemental Table 2. *Longitudinal Changes across Implementation Periods for Skills, Informational Resources, Professional Role & Identity, Self-efficacy, Organizational Context & Resources, and Positive and Negative Emotions based on the Determinants of Implementation Behavior Questionnaire (DIBQ)*

|  | Pre-  PEACHES |  | Post-  Workshop |  | Post-  PEACHES |  |  | | | | | | | | |
| --- | --- | --- | --- | --- | --- | --- | --- | --- | --- | --- | --- | --- | --- | --- | --- |
|  | *N* = 64 |  | *N* = 64 |  | *N* = 58 |  | Pre-PEACHES v. Post-Workshop | | | |  | Pre-PEACHES v. Post-PEACHES | | | |
|  | *Mean* (*SD)* |  | *Mean* (*SD)* |  | *Mean* (*SD)* |  | *MD* | *% MD* | *t* (*df*) | *p*-value |  | *MD* | *% MD* | *t* (*df*) | *p*-value |
| **Skills** | 2.80 (1.42) |  | 5.19 (0.69) |  | 5.19 (0.76) |  | 2.4 | 49% | 12.02 (60) | < .0001 |  | 2.4 | 48% | 11.88 (55) | < .0001 |
| I have been trained to start a conversation with clients about anal pleasure and health. | 1.72 (1.70) |  | 5.23 (1.03) |  | 5.48 (0.71) |  | 3.5 | 70% | 13.82 (62) | < .0001 |  | 3.7 | 74% | 15.63 (57) | < .0001 |
| I have practiced ways to respond to client questions about anal pleasure and health. | 2.44 (1.84) |  | 5.38 (0.65) |  | 5.36 (0.74) |  | 2.9 | 58% | 11.62 (62) | < .0001 |  | 2.9 | 57% | 10.97 (57) | < .0001 |
| I know how to talk with clients about anal pleasure and health. | 2.64 (1.75) |  | 5.32 (0.67) |  | 5.28 (0.79) |  | 2.7 | 55% | 12.17 (61) | < .0001 |  | 2.6 | 52% | 11.04 (57) | < .0001 |
| Starting a conversation with clients about anal pleasure and health is easy for me. | 3.45 (1.69) |  | 4.92 (1.12) |  | 4.81 (1.19) |  | 1.5 | 30% | 6.77 (62) | < .0001 |  | 1.3 | 27% | 5.41 (57) | < .0001 |
| Answering client questions about anal pleasure and health is easy for me. | 3.31 (1.84) |  | 5.19 (0.81) |  | 5.03 (1.11) |  | 1.9 | 38% | 7.49 (62) | < .0001 |  | 1.7 | 34% | 6.69 (57) | < .0001 |
| **Positive Emotions** | 4.13 (1.25) |  | 4.92 (0.92) |  | 4.89 (1.02) |  | 0.8 | 16% | 5.23 (60) | < .0001 |  | 0.7 | 14% | 4.36 (54) | < .0001 |
| When discussion turns to anal sex during a healthcare encounter with a client, I feel natural. | 4.05 (1.45) |  | 4.84 (1.01) |  | 4.85 (1.01) |  | 0.8 | 16% | 4.23 (61) | < .0001 |  | 0.8 | 15% | 4.39 (54) | < .0001 |
| When discussion turns to anal sex during a healthcare encounter with a client, I feel at ease. | 3.94 (1.44) |  | 4.89 (1.04) |  | 4.93 (0.98) |  | 1.0 | 19% | 5.32 (61) | < .0001 |  | 1.0 | 19% | 5.01 (54) | < .0001 |
| When discussion turns to anal sex during a healthcare encounter with a client, I feel comfortable. | 4.22 (1.36) |  | 5.02 (0.92) |  | 5.05 (0.88) |  | 0.8 | 16% | 5.06 (61) | < .0001 |  | 0.8 | 16% | 4.46 (55) | < .0001 |
| **Negative Emotions** | 2.65 (1.38) |  | 2.06 (1.39) |  | 2.06 (1.34) |  | -0.5 | -11% | -2.32 (58) | .0237 |  | -0.7 | -14% | -3.58 (52) | .0007 |
| When discussion turns to anal sex during a healthcare encounter with a client, I feel nervous. | 2.27 (1.68) |  | 2.06 (1.72) |  | 2.14 (1.64) |  | -0.2 | -5% | -0.90 (61) | .3717 |  | -0.2 | -4% | -0.84 (56) | .4028 |
| When discussion turns to anal sex during a healthcare encounter with a client, I feel awkward. | 2.14 (1.72) |  | 1.89 (1.57) |  | 1.84 (1.40) |  | -0.3 | -5% | -1.01 (61) | .3174 |  | -0.3 | -6% | -1.25 (55) | .2150 |
| When discussion turns to anal sex during a healthcare encounter with a client, I worry I'm going to say or do the wrong thing. | 3.06 (1.70) |  | 2.70 (1.75) |  | 2.57 (1.92) |  | -0.3 | -6% | -1.13 (61) | .2639 |  | -0.6 | -11% | -1.87 (55) | .0669 |
| When discussion turns to anal sex during a healthcare encounter with a client, I feel I don't know what to do. | 2.84 (1.58) |  | 1.45 (1.55) |  | 1.50 (1.61) |  | -1.4 | -29% | -5.43 (60) | < .0001 |  | -1.4 | -28% | -4.98 (54) | < .0001 |
| **Professional Role and Identity** | 4.70 (0.97) |  | 5.27 (0.79) |  | 5.26 (0.87) |  | 0.6 | 12% | 4.34 (62) | < .0001 |  | 0.5 | 10% | 3.50 (57) | .0009 |
| Being able to talk with clients about anal health is a part of my work as an HIV worker. | 4.54 (1.47) |  | 5.00 (1.38) |  | 5.29 (0.97) |  | 0.5 | 10% | 2.50 (61) | .0151 |  | 0.6 | 12% | 3.42 (54) | .0012 |
| Being able to talk with clients about anal pleasure is a part of my work as an HIV worker. | 3.97 (1.68) |  | 4.86 (1.49) |  | 5.00 (1.29) |  | 0.9 | 19% | 3.70 (62) | .0005 |  | 1.0 | 20% | 3.96 (55) | .0002 |
| As a health worker, it is my job to increase my own comfort discussing anal pleasure and health. | 5.14 (1.16) |  | 5.70 (0.49) |  | 5.59 (0.59) |  | 0.6 | 11% | 3.69 (61) | .0005 |  | 0.4 | 9% | 2.84 (56) | .0062 |
| It is my responsibility as a health worker to help clients feel more comfortable talking about anal pleasure and health. | 5.28 (0.90) |  | 5.53 (0.87) |  | 5.50 (0.60) |  | 0.2 | 5% | 1.87 (62) | .0664 |  | 0.2 | 4% | 1.66 (57) | .1015 |
| **Self-efficacy**^†^ | 3.94 (1.44) |  | 5.14 (0.74) |  | 4.85 (0.88) |  | 1.2 | 24% | 6.23 (60) | < .0001 |  | 0.9 | 19% | 4.80 (55) | < .0001 |
| I am confident that I can talk about anal pleasure and health even when clients are not motivated. | 3.72 (1.72) |  | 4.95 (1.06) |  | 4.82 (0.93) |  | 1.2 | 24% | 5.38 (62) | < .0001 |  | 1.1 | 22% | 5.14 (56) | < .0001 |
| I am confident that I can talk about anal pleasure and health with clients even when work colleagues do not do this. | 4.30 (1.64) |  | 5.31 (0.81) |  | 5.14 (1.00) |  | 1.0 | 21% | 4.52 (62) | < .0001 |  | 0.9 | 17% | 3.77 (57) | .0004 |
| I am confident that I can discuss anal pleasure and health with clients even when there is little time.^†^ | 3.78 (1.68) |  | 5.22 (0.77) |  | 4.91 (1.01) |  | 1.4 | 29% | 6.59 (62) | < .0001 |  | 1.1 | 22% | 5.19 (57) | < .0001 |
| I am confident that I can start a discussion about anal pleasure and health even when clients are reluctant.^†^ | 3.47 (1.75) |  | 5.08 (0.88) |  | 4.59 (1.23) |  | 1.6 | 32% | 6.94 (62) | < .0001 |  | 1.2 | 23% | 5.16 (57) | < .0001 |
| **Informational Resources** | 3.34 (1.41) |  | 5.60 (0.47) |  | 5.51 (0.53) |  | 2.3 | 46% | 12.52 (61) | < .0001 |  | 2.2 | 44% | 11.21 (56) | < .0001 |
| I can refer clients to accurate and reliable information about anal pleasure and health. | 3.39 (1.80) |  | 5.56 (0.66) |  | 5.53 (0.57) |  | 2.2 | 44% | 9.69 (62) | < .0001 |  | 2.2 | 44% | 9.42 (57) | < .0001 |
| I have the resources that I need to provide clients with accurate and reliable information about anal pleasure and health. | 2.61 (1.78) |  | 5.61 (0.55) |  | 5.47 (0.68) |  | 3.0 | 60% | 13.46 (62) | < .0001 |  | 2.8 | 56% | 11.57 (57) | < .0001 |
| I can find accurate and reliable information to answer client questions about anal pleasure and health. | 3.86 (1.65) |  | 5.63 (0.49) |  | 5.52 (0.60) |  | 1.8 | 36% | 8.45 (62) | < .0001 |  | 1.7 | 34% | 7.52 (57) | < .0001 |
| **Organizational Context and Resources** | 4.15 (0.99) |  | 4.68 (1.13) |  | 4.73 (1.00) |  | 0.5 | 10% | 3.73 (62) | .0004 |  | 0.4 | 9% | 3.17 (56) | .0025 |
| Discussing anal pleasure and health with clients is acceptable within my workplace. | 4.80 (1.37) |  | 5.16 (1.13) |  | 5.26 (0.79) |  | 0.4 | 7% | 1.91 (62) | .0605 |  | 0.4 | 7% | 2.20 (56) | .0317 |
| The leadership team at my work organization supports addressing anal pleasure and health with clients. | 4.61 (1.30) |  | 5.00 (1.30) |  | 4.98 (1.25) |  | 0.4 | 7% | 2.14 (62) | .0366 |  | 0.2 | 5% | 1.14 (56) | .2596 |
| My organization does a good job addressing the anal pleasure and health needs of our clients. | 3.05 (1.44) |  | 3.88 (1.57) |  | 3.93 (1.58) |  | 0.8 | 16% | 3.76 (62) | .0004 |  | 0.8 | 16% | 3.66 (56) | .0006 |

*Note. Comparisons by timepoint are based only on participants with data at both timepoints; statistical tests compare paired responses and may reflect a reduced sample size. All item Likert responses ranged from 1-6, with higher scores indicating greater positive valence: 1 Strongly disagree, 2 Disagree, 3 Somewhat disagree, 4 Somewhat agree, 5 Agree, 6 Strongly agree; % MD = MD/(Scale Range [5]).*

*^†^ Significant differences between Post-Workshop and Post-Intervention for Self-efficacy [MD = -0.36, % MD = -7%, t(57) = -3.05, p = .0034] driven by these items (in order of appearance): MD = -0.34, %MD = -7%, t(57) = -2.42, p = .0187; MD = -0.55, %MD = -11%, t(57) = -3.63, p = .0006.*

# Supplemental Table 3. *Longitudinal Differences across Implementation Periods for Ease/Confidence Offering Social Support based on the Medical Outcomes Study Social Support Survey (MOS-SSS) adapted subscale*

|  | Pre-  PEACHES |  | Post-  Workshop |  | Post-  PEACHES |  |  | | | | | | | | |
| --- | --- | --- | --- | --- | --- | --- | --- | --- | --- | --- | --- | --- | --- | --- | --- |
|  | *N* = 65 |  | *N* = 64 |  | *N* = 59 |  | Pre-PEACHES v. Post-Workshop | | | |  | Pre-PEACHES v. Post-PEACHES | | | |
|  | *Mean* (*SD*) |  | *Mean* (*SD*) |  | *Mean* (*SD*) |  | *MD* | *% MD* | *t* (*df*) | p-value |  | *MD* | *% MD* | *t* (*df*) | p-value |
| How easy would it be for you to support clients in the following ways? | 4.03 (0.93) |  | 4.41 (0.52) |  | 4.35 (0.56) |  | 0.37 | 9% | 3.35 (63) | .0014 |  | 0.32 | 8% | 2.87 (58) | .0058 |
| Be someone to count on to listen when a client needs to talk about anal sex. | 4.40 (0.83) |  | 4.63 (0.55) |  | 4.56 (0.62) |  | 0.20 | 5% | 2.27 (63) | .0268 |  | 0.12 | 3% | 1.19 (58) | .2399 |
| Be someone to give information to help a client understand a situation involving anal sex. | 3.98 (1.15) |  | 4.38 (0.65) |  | 4.37 (0.72) |  | 0.38 | 10% | 2.32 (63) | .0234 |  | 0.39 | 10% | 2.81 (58) | .0068 |
| Be someone to give a client good advice about a crisis related to anal sex. | 3.86 (1.17) |  | 4.25 (0.73) |  | 4.19 (0.78) |  | 0.38 | 10% | 2.58 (63) | .0121 |  | 0.34 | 9% | 2.26 (58) | .0278 |
| Be someone a client could confide in or talk to about anal sex. | 4.38 (0.88) |  | 4.59 (0.56) |  | 4.61 (0.56) |  | 0.20 | 5% | 1.89 (63) | .0628 |  | 0.20 | 5% | 1.76 (58) | .0832 |
| Be someone whose advice about anal sex a client would really want.† | 3.68 (1.28) |  | 4.28 (0.65) |  | 4.10 (0.78) |  | 0.59 | 15% | 3.82 (63) | .0003 |  | 0.42 | 11% | 2.45 (58) | .0173 |
| Be someone a client could share their most private worries and fears with about anal sex. | 4.38 (0.86) |  | 4.52 (0.59) |  | 4.49 (0.60) |  | 0.11 | 3% | 0.98 (63) | .3309 |  | 0.08 | 2% | 0.76 (58) | .4505 |
| Be someone to turn to for suggestions about how to deal with a personal problem with anal sex. | 3.83 (1.21) |  | 4.38 (0.65) |  | 4.25 (0.78) |  | 0.52 | 13% | 3.18 (63) | .0023 |  | 0.44 | 11% | 2.84 (58) | .0063 |
| Be someone who understands clients' problems with anal sex. | 3.71 (1.28) |  | 4.28 (0.74) |  | 4.20 (0.92) |  | 0.56 | 14% | 3.86 (63) | .0003 |  | 0.54 | 14% | 3.10 (58) | .0030 |

*Note. Comparisons by timepoint are based only on participants with data at both timepoints; statistical tests compare paired responses and may reflect a reduced sample size. All item Likert responses are reverse-coded to range from 1-5, with higher scores indicating greater ease: 1 Very Difficult, 2 Difficult, 3 Neither Easy nor Difficult, 4 Easy, 5 Very Easy; % MD = MD/(Scale Range [4]).*

*^†^Significant difference between Post-Workshop and Post-Intervention [MD = -.022, % MD = -6%, t(58) = -2.03, p = .0465].*
